# Hypoxia and Hypoxia‐Reoxygenation Potentiate *Helicobacter pylori* Infection and Gastric Epithelial Cell Proliferation

**DOI:** 10.1002/cam4.70860

**Published:** 2025-05-05

**Authors:** Indrajit Poirah, Soumyadeep Chakraborty, Pratyush Kumar Padhan, Ashish Kumar Mishra, Debashish Chakraborty, Pragyesh Dixit, Supriya Samal, Niranjan Rout, Shivaram Prasad Singh, Gautam Nath, Duane T. Smoot, Hassan Ashktorab, Asima Bhattacharyya

**Affiliations:** ^1^ School of Biological Sciences, National Institute of Science Education and Research (NISER) Bhubaneswar An OCC of Homi Bhabha National Institute Odisha India; ^2^ Dioscuri Centre for Physics and Chemistry of Bacteria Institute of Physical Chemistry‐Polish Academy of Sciences Warsaw Poland; ^3^ Digestive Diseases Centre Beam Diagnostics Building Odisha India; ^4^ Department of Gastroenterology Acharya Harihar Post Graduate Institute of Cancer Cuttack India; ^5^ Department of Medicine Meharry Medical Center Nashville TN USA; ^6^ Department of Medicine Howard University Washington DC USA; ^7^ Centre for Interdisciplinary Sciences (CIS), NISER An OCC of Homi Bhabha National Institute Odisha India

**Keywords:** carcinoembryonic antigen, *Helicobacter pylori*, hypoxia, hypoxia‐inducible factor 1, hypoxia‐reoxygenation, NADPH oxidase 4, reactive oxygen species

## Abstract

**Introduction:**

The gastric epithelium experiences intermittent hypoxia due to various physiological and pathological conditions. However, the impact of hypoxia and hypoxia‐reoxygenation of gastric epithelial cells (GECs) on Helicobacter pylori‐mediated gastric cancer (GC) has never been investigated. Carcinoembryonic antigen‐related cell adhesion molecules (CEACAMs) facilitate H. pylori adhesion onto GECs. We evaluated the effect of hypoxia and hypoxia‐reoxygenation on CEACAM6‐mediated H. pylori binding, infection, reactive oxygen species (ROS) generation, and GEC proliferation.

**Methods:**

Hypoxia‐inducible factor 1 (HIF1α) and CEACAM6 levels were assessed in various GECs. ROS were measured using 2',7'‐dichlorofluorescin diacetate (DCFDA). Bioinformatics analyses were performed to identify the most prominent stomach adenocarcinoma (STAD)‐associated NADPH oxidase (NOX) followed by validation by overexpression/suppression studies and western blotting. GC biopsies were examined by immunofluorescence microscopy. Hypoxia‐exposed, reoxygenated, or control cells were compared for ROS generation and H. pylori infection. MTT assay determined cell proliferation.

**Results and Conclusions:**

Hypoxia and HIF1 mediated upregulation of CEACAM6 in GECs. CEACAM6 significantly promoted ROS generation by inducing NOX4 in hypoxic GECs. HIF1α, CEACAM6, and NOX4 upregulation was detected in gastritis and GC tissues. H. pylori infection significantly increased in hypoxia‐exposed GECs as compared to normoxic GECs. Infection of hypoxia‐reoxygenated GECs also resulted in significantly increased CEACAM6 and NOX4‐mediated ROS generation compared to normoxic GECs. In addition, adhesion of H. pylori, cytotoxin‐associated gene A (CagA) translocation, and GEC proliferation were significantly enhanced in hypoxia‐reoxygenated GECs. Collectively, this study established that hypoxia and hypoxia‐reoxygenation of GECs facilitate H. pylori infection and infection‐mediated GEC proliferation.

## Graphical abstract

Hypoxia and hypoxia‐reoxygenation upregulate CEACAM6. It potentiates ROS generation via NOX4. Moreover, CEACAM6 upregulation facilitates *H. pylori* adhesion to hypoxic gastric epithelial cells, establishes infection leading to the aggressiveness of gastric cancer.

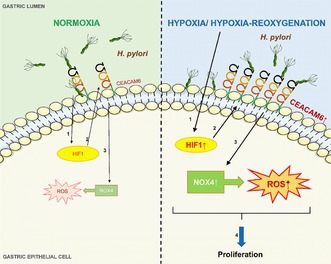

**Abbreviations**
Cag PAI: cytotoxin‐associated gene pathogenicity island
CagA: cytotoxin‐associated gene A
CEACAM: carcinoembryonic antigen‐related cell adhesion molecule
CFU: colony‐forming unit
C‐SRC: cellular sarcoma kinase
DAPI: 4′,6‐diamidino‐2‐phenylindoledihydrochloride
DCFDA: 2,7‐dichlorofluoroscein diacetate
DPBS: Dulbecco's phosphate buffer saline
EDTA: ethylene diamine tetra acetic acid
EMT: epithelial to mesenchymal transition
FAK: focal adhesion kinase
GC: gastric cancer
GEC: gastric epithelial cell
GPI: glycophosphatidylinositol

*H. pylori*

:
*Helicobacter pylori*

H_2_O_2_
: hydrogen peroxide
HIF1: hypoxia‐inducible factor 1
LOX: lysyl oxidase
MAPK: mitogen‐activated protein kinase
MFI: mean fluorescence intensity
MOI: multiplicity of infection
MTT: 3‐(4, 5‐dimethylthiazolyl‐2)‐2, 5‐diphenyltetrazolium bromide
NOX: NADPH oxidase
PBS: phosphate‐buffered saline
PI3K/Akt: phosphoinositide‐3‐kinase‐protein kinase B/Akt kinase
ROS: reactive oxygen species
SEAP: secretory alkaline phosphatase
STR: short tandem repeat
TSA: trypticase soy agar

## 1 Introduction

Gastric tissue can experience temporary, prolonged, intermittent, or cyclical hypoxia. Hypoxia of the gastric mucosa is also a common signature of “aging gastropathy” [1]. Despite some understanding, the effect of hypoxia on *H. pylori* ‐mediated gastric carcinogenesis is mainly unexplored. *H. pylori* infection remains one of the major causes of GC in the developing world [2]. Since living at high altitude increases the occurrence of GC with a positive correlation with *H. pylori* infection and high gastric epithelial HIF1 level [3, 4, 5], we hypothesize that hypoxia exposure might facilitate *H. pylori* ‐mediated carcinogenic events.

Hypoxia promotes tumorigenesis by regulating several cellular functions [6], often driven by deregulated oncogenes. One such oncogene and transcription factor is HIF1 which promotes GC and several other cancers [7, 8, 9, 10, 11, 12]. HIF1α subunit is oxygen‐labile and the β subunit is constitutively expressed. Upon stabilization of the α subunit in hypoxia, the α‐β dimer forms the active transcription factor HIF1 which activates genes that help cells overcome the hypoxic stress. CEACAMs belong to an immunoglobulin‐like large family of membrane‐anchored cell surface glycoproteins involved in cell–cell as well as host–pathogen interactions [13]. Epithelial CEACAMs such as CEACAM1, CEACAM5, CEACAM6, and CEACAM7 are expressed in the human gastric epithelium under nonpathological conditions. However, their levels increase significantly in GC [14]. CEACAMs are significant players in *H. pylori* infection too. CEACAMs facilitate the binding of *H. pylori* to GECs and help in the translocation of the *H. pylori* virulence factor cytotoxin‐associated gene A (CagA) [15]. Interestingly, CagA induces CEACAM6 expression in GECs [16]. The accumulation of ROS in the *H. pylori‐*infected GECs stabilizes HIF1α, promotes oxidative stress, and initiates chronic inflammation [9, 17, 18, 19]. Inflammation induces various mucosa‐associated pathogenic events leading to GC [20]. Although HIF1‐regulated *CEACAM6* expression in Crohn's disease facilitates *Escherichia coli* infection in intestinal epithelial cells, the effect of hypoxia on CEACAM6 and its relevance in the pathogenesis of GC is unclear [21]. NOX‐mediated hydrogen peroxide (H_2_O_2_) generation remains one of the major contributors to *H. pylori* ‐mediated gastric carcinogenesis [22]. Notably, NOX4 is transcriptionally upregulated in the gastrointestinal tract experiencing hypoxia and enhances H_2_O_2_ generation [23]. GC tissues show significantly higher expression of NOX4 when compared with the normal gastric epithelium, and the protein is correlated with poor prognosis [24, 25]. Although CEACAM1 knockdown promotes NOX4 in endothelial cells, the role of CEACAMs in the regulation of NOXs in hypoxia‐exposed and *H. pylori* ‐infected GECs is completely elusive [26].

There are few reports highlighting the status of CEACAM6 as a GC marker; however, no current studies reveal the importance of studying CEACAM6 in the context of a hypoxic microenvironment in GC. Since an earlier report on rat gastric mucosa has shown that hypoxia‐regulated gene expressions do not alter with hypoxia‐reoxygenation [27], this study explored the status of HIF1, CEACAM6, and NOX4 after *H. pylori* infection in GECs exposed to hypoxia and hypoxia‐reoxygenation.

## 2 Materials and Methods

### 2.1 Gastric Epithelial Cell Lines, Culture Conditions, Hypoxia or Hypoxia‐Reoxygenation Exposure, and *H. pylori* Infection

Before experimentation, it was imperative to understand the reference points of “normoxia” and “hypoxia” for GECs. While the inhaled air contains ~21% O_2_, various human tissue O_2_ levels remain much lower than that [28]. The human stomach lumen contains 14%–19% O_2_ [29]. A healthy human stomach normally experiences partial pressure of O_2_ between ~60 and 77 mmHg (~8%–10% O_2_) from the luminal side to the muscular wall [30, 31]. However, postprandial hyperemia allows increased O_2_ supply to the stomach tissue and, after each meal, for several hours, the O_2_ supply remains elevated [32]. This indicates that healthy human GECs are tolerant to a broad range of O_2_ concentrations, and lower than 5% O_2_ can be considered hypoxic for GECs. *H. pylori* remains in the gastric lumen and invades the mucosa. To infect GECs with *H. pylori* in vitro, GECs are conventionally incubated in the presence or absence of the pathogen in a 5% CO_2_‐containing humidified incubator where O_2_% remains 16%. However, in vitro experiments maintaining 5% CO_2_ show that the viability of *H. pylori* gradually declines and apoptosis ensues in infected macrophages when the O_2_% is reduced from 20% to 1% [33]. Considering the O_2_ tolerance range of GECs and *H. pylori* viability issues, 16% O_2_ was designated as normoxia (indicated as N in Figures) and 3% O_2_ (indicated as H in Figures) as hypoxia for GECs.

AGS cell line (ATCC), AGS‐derived stable cells (described in the next section), MKN45 cells (sourced from Dr. Sheila E. Crowe's lab, UVA, USA), and non‐malignant GEC‐ HFE145 (sourced from Dr. Hassan Ashktorab's lab, Howard University, USA) were cultured and maintained in 10% heat‐inactivated FBS‐supplemented RPMI1640 (HIMEDIA, Nashik, India) at 16% O_2_ and 5% CO_2_ in a humidified CO_2_ incubator maintaining 37°C. Cells were authenticated by short tandem repeat (STR) profiling. *H. pylori* cag PAI (+) strain 26695 was maintained and cultured following standard protocols [34, 35]. For hypoxia‐reoxygenation experiments, after incubating for 12 h in normoxia or hypoxia, GECs were infected with *H. pylori* at a multiplicity of infection (MOI) 50 for 12 h or were left uninfected in normoxia. To induce hypoxia, cells were incubated at 37°C, 5% CO_2_, and 3% O_2_ in a Whitley H35 Hypoxystation (Don Whitley Scientific Ltd., West Yorkshire, United Kingdom).

### 2.2 Transfections and Generation of Stable Cells

Human *CEACAM6*‐pdKCR‐neo construct, pCMV6‐Xl5‐*HIF1α* (Origene Technologies, MD, USA), human *NOX4* (courtesy: Karl‐Heinz Krause, Addgene plasmid #69352) overexpression plasmids and empty vectors were used in this study [36, 37]. Human *CEACAM6* siRNA, *NOX4* siRNA, and control siRNA were purchased from Santa Cruz Biotechnology (TX, USA). After the transfection of constructs, all stable overexpression and knockdown cells (in AGS) were generated using standard protocols [38, 39, 40]. Stable *HIF1α* knockdown AGS cells were used in some experiments [41]. Lipofectamine3000 (Invitrogen, CA, USA) was used for all transfections according to the manufacturer's protocol.

### 2.3 Human Gastritis and GC Biopsy Sample Collection

Human GC (*n* = 3) and gastritis (*n* = 3) biopsy specimens were collected from consenting patients and were included in the study after testing for urease positivity. Adjacent non‐neoplastic/uninvolved gastric tissues were used as matched controls. Patient confidentiality was protected. Patients undergoing treatment for *H. pylori* eradication were excluded from this study. The collection protocol was approved by the ethics committee of NISER and complied with the Helsinki Declaration (2013) of the World Medical Association.

### 2.4 Gene Expression and Related Analysis

The Gene Expression Profiling Interactive Analysis 2 (GEPIA2) database (http://gepia2.cancer‐pku.cn/#index) includes RNA sequencing and expression data from tumor samples, malignant cancers, and normal tissues [42]. The correlation between *HIF1α* and various *NOX* family mRNAs as well as between *CEACAM6* and *NOX4* mRNAs was determined using the expression data from the “correlation analysis” module of GEPIA2 utilizing The Cancer Genome Atlas (TCGA) tumor and normal datasets. Pearson's correlation analyses were performed to determine the correlation coefficient *R* and the *p*‐value (data accessed on May 5, 2023). Gene expression was normalized with *TUBA4A*, and the generated scatter plots displayed the data as log2 transcripts per million (TPM).

The “Expression analysis – Box Plot” module of the GEPIA2 was used to analyze the expression of STAD and normal tissues of the TCGA database (settings used: “match TCGA normal data,” *p*‐value cutoff = 0.01 or at 0.05 and fold change ILog_2_FCI cutoff = 1). Violin plots were generated to understand the *NOX4* expression at various pathological stages of STAD (stage I, II, III, and IV) using the “Expression Analysis‐Stage Plot” module of GEPIA2. Both the box and violin plots applied the log2(TPM + 1) transformed expression data (analyses were done on May 5, 2023).

The interactive web portal UALCAN performs pan‐cancer gene expression analysis based on clinical data from 31 cancers and level‐3 TCGA RNA‐seq data [43]. To further study the correlation between *NOX4* gene expression and the clinicopathological features of GC, subgroup analyses were performed using the UALCAN database (http://ualcan.path.uab.edu) on May 5, 2023. The correlation of *CEACAM6* or *NOX4* expression and various parameters such as age, gender, and race in STAD were analyzed through UALCAN using the TCGA samples. *p* ≤ 0.05 was considered statistically significant. “Multiple genes comparison” for *HIF1α*, *CEACAM6*, and *NOX4* was performed in GEPIA2 selecting “Matched TCGA normal data” and “Yes” for the log scale.

*CEACAM6* and *NOX4* mRNA expression across various types of STAD tumors was determined from “tumor type” analysis available from the cBioPortal for Cancer Genomics (www.cbioportal.org) [44, 45]. From the TCGA pan‐cancer atlas, 440 stomach adenocarcinoma samples were selected for analysis (the date of data acquisition was May 5, 2023). mRNA versus Dx Plots of tumor type versus mRNA expression were generated for *CEACAM6* and *NOX4* based on the batch‐normalized expression obtained from Illumina HiSeq_RNASeQV2 using the RNA‐seq expectation–maximization (RSEM) tool. Plots showed 371 and 407 samples (for NOX4 and CEACAM6, respectively) with data in both profiles (axes).

### 2.5 Western Blotting

GECs were lysed to obtain total cell lysates, separated by SDS‐PAGE, and electrophoretically transferred to PVDF membranes following standard protocols [46]. CEACAM6 (Novus Biologicals, CO, USA), NOX4 (Novus Biologicals), Hif1α (Abcam, MA, USA), CagA (Santa Cruz Biotechnology), and α‐tubulin (Biobharati, WB, India) primary antibodies were used to probe electro‐blotted membranes. Chemiluminescence was detected with the SuperSignal West Femto kit (Thermo Fisher Scientific, IL, USA). Immunoblot images were captured utilizing BioRad Chemidoc XRS+ (Bio‐Rad Laboratories, CA, USA). Densitometry was performed using ImageLab (Bio‐Rad).

### 2.6 Immunofluorescence Microscopy

AGS cells were plated on glass coverslips and exposed to normoxia and/or hypoxia followed by *H. pylori* infection or no infection. Cells were fixed using 4% paraformaldehyde. Cells were permeabilized in 0.1% Triton X‐100 in PBS followed by blocking in 5% bovine serum albumin solution at RT for 1 h followed by overnight incubation with the specific primary antibodies at 4°C, at manufacturer‐recommended dilutions. Corresponding Alexa Fluor‐tagged secondary antibodies (Invitrogen, OR, USA) were used, followed by nuclear staining with 4',6‐diamidino‐2‐phenylindole dihydrochloride (DAPI) (Invitrogen). Imaging was performed with either a fluorescence (Nikon Eclipse TiU/Eclipse Ni‐E, Nikon, Japan) or a confocal (Leica DMi8, Leica, Germany) microscope using Leica Application Suite X software. Image processing and analysis were performed using NIS Advanced Research software (Nikon) or Fiji (ImageJ) [47].

### 2.7 Observation of the Hummingbird Phenotype

AGS cells were exposed to hypoxia/normoxia and *H. pylori* infection, washed with 1× PBS, and imaged under the 40× or 20× objective of a bright field microscope (Nikon) equipped with the digital monochrome camera DS Qi2 (Nikon). A cell was considered to bear the hummingbird phenotype, a classical signature of *H. pylori* infection, when the ratio of the cell's longest projection to the shortest diameter was greater than 2 [48]. At least 50 cells were counted from five fields. The percentage of hummingbird cells was calculated using Fiji, and statistical significance was calculated using GraphPad Prism (GraphPad, CA, USA).

### 2.8 *H. pylori* Adhesion Assay Using Confocal Microscopy

AGS cells were plated on glass coverslips and exposed to normoxia and/or hypoxia followed by *H. pylori* infection or no infection in normoxic conditions. Cells were processed for immunofluorescence/confocal microscopy as per the standard protocol; nuclei were stained with DAPI and cells were stained to detect CEACAM6. Adhesion of *H. pylori* to AGS cells was visualized using the anti‐ *H. pylori* antibody (Abcam). Imaging and analysis were performed as described before.

### 2.9 Counting of *H. pylori* Colony‐Forming Units (CFU)

After *H. pylori* infection of normoxic, hypoxic, or hypoxia‐reoxygenated CEACAM6‐stable cells, the cells were washed twice with PBS and treated with 0.25% trypsin +0.02% EDTA in DPBS solution (HIMEDIA). Cell suspensions were serially diluted, plated on Trypticase Soy Agar (TSA) plates (Becton Dickinson, NJ, USA), and cultured in microaerophilic condition for 3 days to count viable *H. pylori* colonies expressed as CFU. Graphs were generated with data from three independent experiments.

### 2.10 Detection of HopQ–CEACAM6 Interaction by Co‐Immunoprecipitation

*H. pylori* was cultured in 100 ml Brucella broth overnight and harvested upon reaching an OD_600_ value of 1. The bacterial pellet was resuspended in 10 mM Tris–HCl (pH 7.5) + 2% Triton X‐100 (dissolved in 10 mM Tris base [pH 7.5]) and lysed by sonication. The outer membrane proteins were extracted following an established protocol [49]. Empty vector‐transfected as well as *CEACAM6*‐stable AGS cells were grown in 100 mm dishes and subjected to either a normoxic or a hypoxic environment. After 12 h, cells were lysed using RIPA buffer (HiMedia) supplemented with Roche complete protease inhibitor cocktail (Roche, Germany) followed by protein estimation of bacterial lysate and cell lysate. Cell lysates were incubated with bacterial outer membrane lysate in Tris EDTA‐NaCl (TEN) buffer for 6 h at 4°C on a nutating rotator. Following this, 3.5 μg of rabbit monoclonal CEACAM6 antibody (Abcam) was added to each microcentrifuge along with an equal amount of rabbit IgG (Cell Signaling Technology, MA, USA) in its respective microcentrifuge tube. Reaction cocktails were incubated overnight at 4°C on a nutating rotator. Next, microcentrifuges were centrifuged briefly at 2350 *g* for 5 min at 4^o^C,  7.5 μg Protein A/G PLUS‐Agarose (Santa Cruz Biotechnology) was added for the pulldown and rotated for another 3 h. After the pulldown, samples were centrifuged at 850 *g* for 5 min at 4^o^C, the supernatant was discarded and beads were washed twice in ice‐cold PBS followed by boiling at 100°C for 8 min in 1× sample buffer. Lysates were analyzed by western blotting.

### 2.11 Detection of ROS

AGS cells were treated as mentioned earlier. Spent media was removed with 1× PBS wash. Cells were treated with 1 μM 2, 7‐dichlorodihydrofluorescein diacetate (DCFDA; Sigma‐Aldrich, MA, USA), nuclei were counterstained with DAPI, and ROS were measured as already reported [34]. The mean fluorescence intensity (MFI) indicating ROS level was calculated from three separate fields from three biological repeats.

### 2.12 MTT Assay

The proliferation potential of GECs was assessed using an MTT assay kit (EZcount MTT cell assay kit, Himedia) following the manufacturer's protocol.

### 2.13 Statistical Analyses

GraphPad Prism 9.5.1 software (San Diego, CA, USA) was used for statistical analyses. *p* ≤ 0.05 was considered statistically significant. Statistical significance was determined using either the Student's *t*‐test or one‐way and two‐way ANOVA followed by Tukey's post hoc test.

## 3 Results

### 3.1 Hypoxia Upregulates CEACAM6 in GECs

**FIGURE 1:**
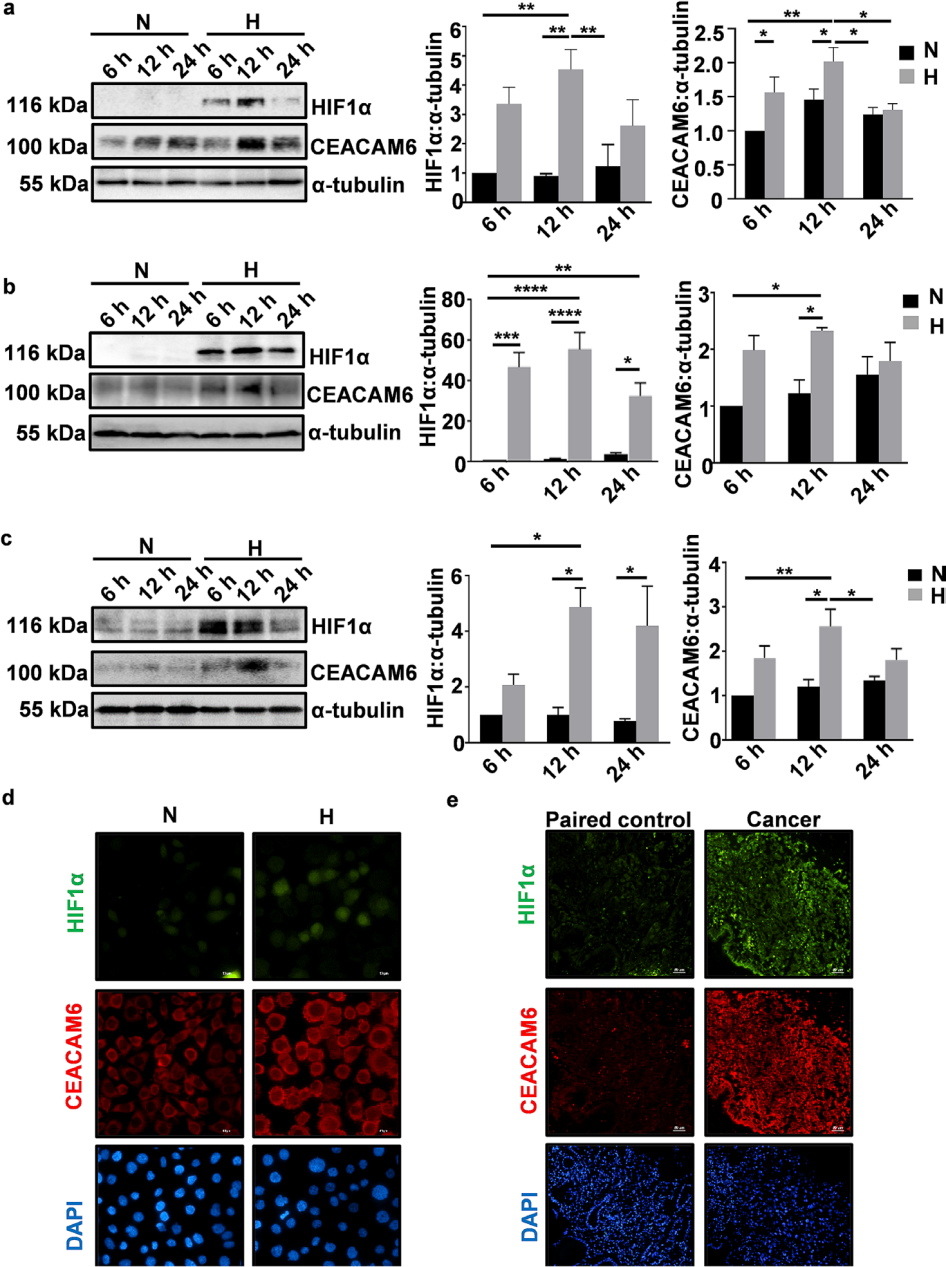
Hypoxia induces CEACAM6. Western blotting of whole cell lysates of AGS (a), MKN45 (b), and HFE145 (c) cells kept at normoxia or hypoxia (=H, 3% O_2_) showing levels of HIF1α and CEACAM6 at 6, 12, and 24 h. α‐tubulin was used as loading control. Bar graphs indicated a significant increase in CEACAM6 level at 12‐h time point. (d) A representative (*n* = 3) immunofluorescence micrograph showing elevated levels of HIF1α (green) and CEACAM6 (red) in AGS cells at 12 h of hypoxia (3% O_2_) exposure. Objective used = 40×, scale bar = 50 μm. (e) A representative (*n* = 3) immunofluorescence micrograph of human metastatic GC biopsy tissue sample showing the status of HIF1α (red) and CEACAM6 (green). Nuclei were stained for DAPI (blue). Tissues were sectioned at 5 μm thickness. Images were captured using 20× objective and scale bars = 50 μm. Graphs = mean ± SEM. Statistical significance was determined using two‐way ANOVA followed by Tukey's post hoc analysis (*n* = 3). *p < 0.05; **p < 0.01; ***p  < 0.001; ****p < 0.0001.

To identify the effect of hypoxia on gastric epithelial CEACAM6 protein and determine the optimal time point of CEACAM6 upregulation at 3% O_2_, AGS cells were exposed to 3% O_2_ for 6, 12, and 24 h. From a representative western blot (*n* = 3), a significant increase in CEACAM6 level was noticed at 6 and 12 h (Figure 1a). To find out whether metastatic GC cells (MKN45) respond in the same manner as gastric adenocarcinoma cells (AGS), MKN45 cells were incubated in 3% O_2_ for 6, 12, and 24 h. MKN45 cells showed the trend of a significant increase in CEACAM6 only at 12 h of 3% O_2_ exposure (Figure 1b). The effect of hypoxia exposure on noncancerous HFE145 cells was assessed by incubating cells at 3% O_2_ for various durations (6, 12, and 24 h). A significant CEACAM6 increase was observed in HFE145 cells at 12 h hypoxia (Figure 1c). Hence, all future hypoxia treatments were performed at 3% O_2_ for 12 h. In all of the above‐mentioned results, the HIF1α level decreased at 24 h as compared with the 12 h time point which corroborated earlier reports that prolonged hypoxia reactivates prolyl hydroxylases and causes instability of *HIF1α* mRNA [50]. Fluorescence microscopy of hypoxic and normoxic AGS cells further established hypoxia as a potent inducer of CEACAM6 (Figure 1d). Comparison of metastatic GC (*n* = 3) with paired non‐cancer gastric tissues revealed upregulation of CEACAM6 and HIF1α in the GC tissues (Figure 1e).

### 3.2 CEACAM6 Upregulation in Hypoxia Is HIF1α‐Dependent

**FIGURE 2:**
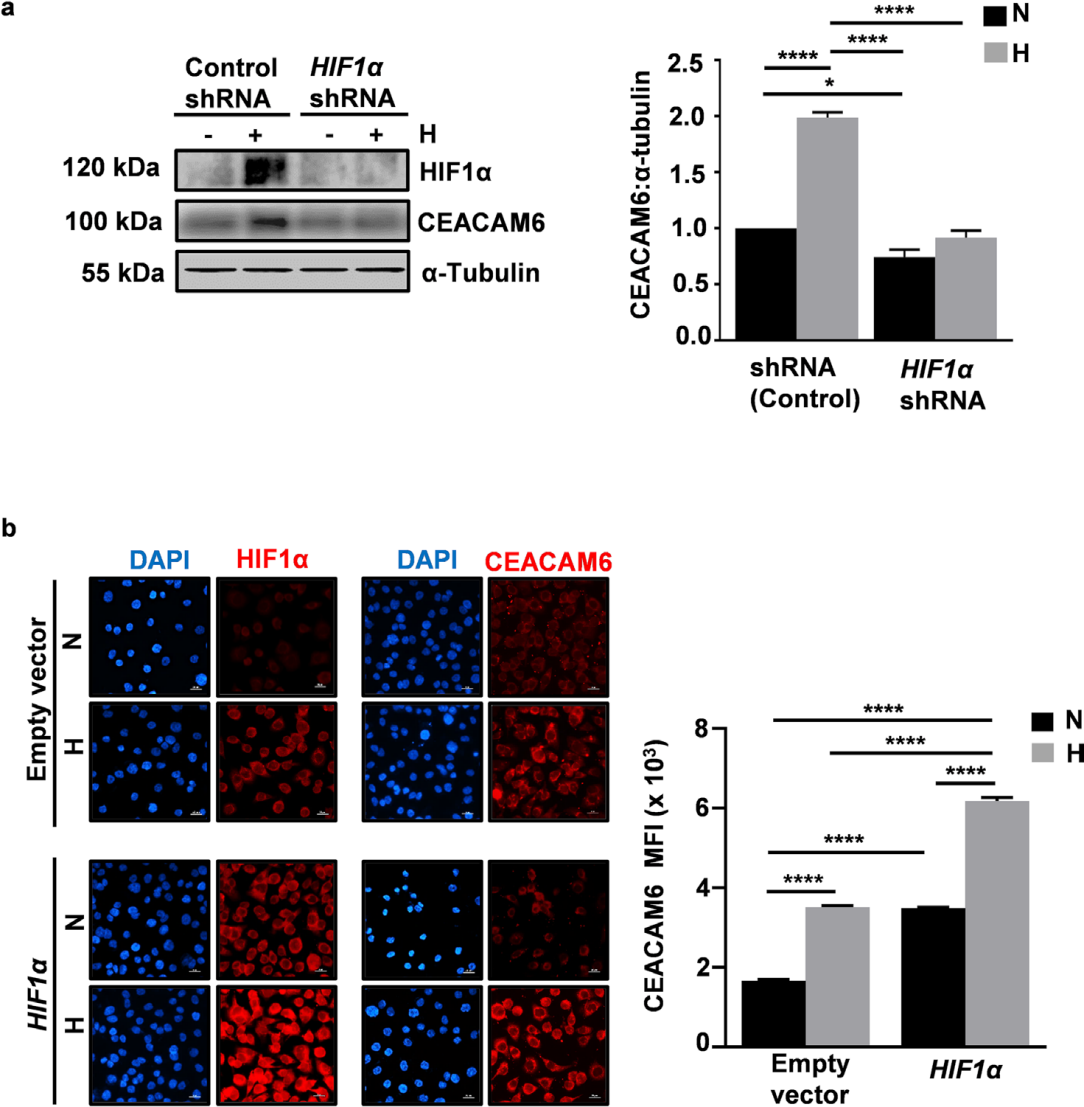
HIF1α upregulates CEACAM6 in hypoxic GECs. (a) Knockdown of HIF1α decreased hypoxia‐induced CEACAM6 protein level in AGS cells as detected by western blot. Whole cell lysates of HIF1α shRNA expressing as well as control shRNA‐expressing stable AGS cells, kept under normoxic and hypoxic conditions for 12 h, showed decreased levels of HIF1α as well as CEACAM6. Bar graphs showed a significant reduction in CEACAM6:α‐tubulin ratio in normoxia as well as hypoxia. (b) Representative micrographs showing CEACAM6 upregulation in HIF1α stably expressing AGS cells at 12 h hypoxia. The nuclei were stained with DAPI. Scale bar = 20 μm, =60×. Bar graph indicated significant changes in the mean fluorescence intensity. Hypoxia = 3% O_2_. All graphical data indicated mean ± SEM. Two‐way ANOVA was applied to determine statistical significance, and the results were corrected for multiple comparisons using Tukey's post hoc analysis. *n* = 3, *p < 0.05; ****p < 0.0001.

The association of CEACAM6 upregulation with hypoxia became evident from Figure 1. To find out the relation of HIF1α with CEACAM6 expression, AGS cells were stably transfected with *HIF1α* shRNA and exposed to hypoxia. In comparison to the control shRNA‐expressing hypoxic cells, *HIF1α* shRNA‐transfected hypoxic cells had significantly less HIF1α and CEACAM6 protein levels (Figure 2a). To further assess the effect of *HIF1α* overexpression on CEACAM6 level, *HIF1α* or the empty vector was stably transfected in AGS cells and cells were exposed to either normoxia or hypoxia. Fluorescence microscopy confirmed that CEACAM6 was significantly upregulated in *HIF1α* stably transfected AGS cells after hypoxia exposure (Figure 2b).

### 3.3 CEACAM6 Expression Is Correlated With NOX4 in Hypoxic GC Tissue

**FIGURE 3:**
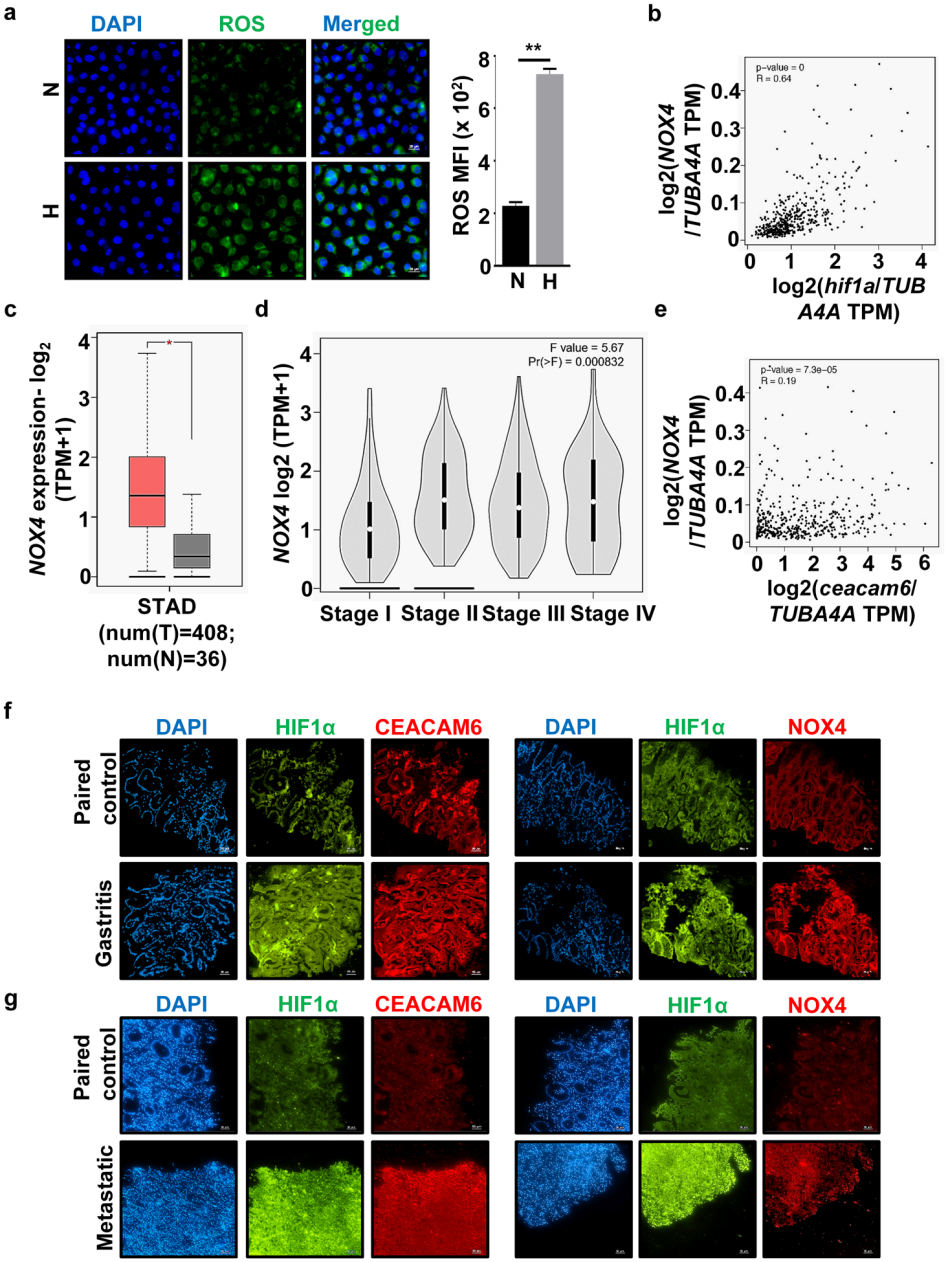
Hypoxia and HIF1α upregulate CEACAM6 and NOX4 in hypoxic GECs, gastritis as well as GC samples. (a) Immunofluorescence microscopy images showing enhanced ROS production in AGS cells under hypoxia. The mean fluorescence intensity (MFI) graph denoted the significant difference in ROS generation between normoxia and hypoxia. Graphical data indicated mean ± SEM. Paired *t*‐test was performed to determine statistical significance. *n* = 3, **p < 0.01. (b) Correlation analysis performed in GEPIA2 showed a positive Pearson correlation coefficient between *HIF1α* and *NOX4* gene expression normalized to *TUBA4A* (*p* = 0, *R* = 0.64). (c) Box plot representation of *NOX4* expression in STAD tumor and normal samples (red = tumor, gray = normal) showed a significant *NOX4* upregulation in tumor samples (num[T] = 408, num[N] = 36, *p* value cutoff was 0.01). (d) Pathological stage plot showed a high association of *NOX4* with the four stages of STAD tumor (*F* value = 5.67, Pr(>*F*) = 0.000832). (e) GEPIA2 correlation analysis showed a positive Pearson correlation coefficient between *CEACAM6* and *NOX4* (*p* = 7.3e‐05, *R* = 0.19). (f) Immunofluorescence micrographs of human gastritis biopsy tissue sample showing the status of HIF1α (green), CEACAM6 (red), and NOX4 (red) (*n* = 3). (g) Human metastatic GC biopsy tissue and their paired normal tissues (*n* = 3) showing enhanced expression of HIF1α (green), CEACAM6 (red), and NOX4 (red) in GC samples. Nuclei were stained for DAPI (blue). Graphical representation showing significant changes in the levels of HIF1α, CEACAM6, and NOX4 are present in Figure S3. Tissues were sectioned at 5 μm thickness. Images were captured using 20× objective and scale bars = 50 μm. In panel c, *indicates significance.

GC strongly correlates with ROS generation [9, 51]. Our data revealed that hypoxia exposure also significantly enhanced ROS levels in AGS cells (Figure 3a). Since ROS‐generating NOX enzymes are upregulated in hypoxia, and *NOX4* knockdown can block GC cell proliferation [52, 53], next we determined the correlation of the expression of various *NOX* family members with *HIF1α* expression in STAD tumor using GEPIA2. *NOX2* and *NOX4* showed the most positive correlation (*p* < 0.05 and *R*‐value > 0.6) with *HIF1α* (Figure S1a and Figure 3b, respectively) while the other ROS‐generators cyclooxygenase 2, cytochrome P450 1A2, lipoxygenase, myeloperoxidase, and xanthine dehydrogenase did not show good correlation with *HIF1α* (Figure S1b–f). *NOX4* was significantly upregulated in STAD tumor when compared with STAD normal, as revealed by the box plot analysis {num[T] = 408, num[N] = 36, *p*‐value cutoff was set at 0.01} (Figure 3c). *NOX2* expression did not show a significant difference between STAD tumor and STAD normal at the same settings (Figure S1g) and at *p*‐value cutoff set at 0.05 as well (Figure S1h). A multiple gene comparisin STAD tissues by GEPIA2 showed higher tumor‐specific expression of *HIF1a*, *CEACAM6*, and *NOX4* as compared to the STAD normal tissue (Figure S1i). Pathological stage plot showed a consistently high association of *NOX4* expression with all stages of STAD (*F* value = 5.67, Pr(>*F*) = 0.000832) (Figure 3d). To find out the correlation between *CEACAM6* and *NOX4* in STAD tissue, Pearson's correlation was determined by using GEPIA2. Although *CEACAM6* was positively correlated with *NOX4* (*p* < 0.05), it *was* a weak correlation (*R* = 0.19) (Figure 3e) The possible reason could be the variations observed in the *CEACAM6* and *NOX4* expression in STAD tissues based on age, sex, and race as determined by the UALCAN analyses (Figure S2a). Across different types of adenocarcinomas of the stomach and even in the same adenocarcinoma type, the expression level of *CEACAM6* and *NOX4* showed huge variations (Figure S2b). Next, the status of HIF1α, CEACAM6, and NOX4 proteins was experimentally validated in normal, gastritis, and adenocarcinoma tissues. Compared to the normal samples, HIF1α, CEACAM6, and NOX4 levels were elevated in gastritis (Figure 3f) and metastasis (Figure 3g) samples (*n* = 3). Quantitative representations of Figure 3f,g are shown in Figure S3.

### 3.4 HIF1α and CEACAM6 Upregulate NOX4 in Hypoxic GECs

**FIGURE 4:**
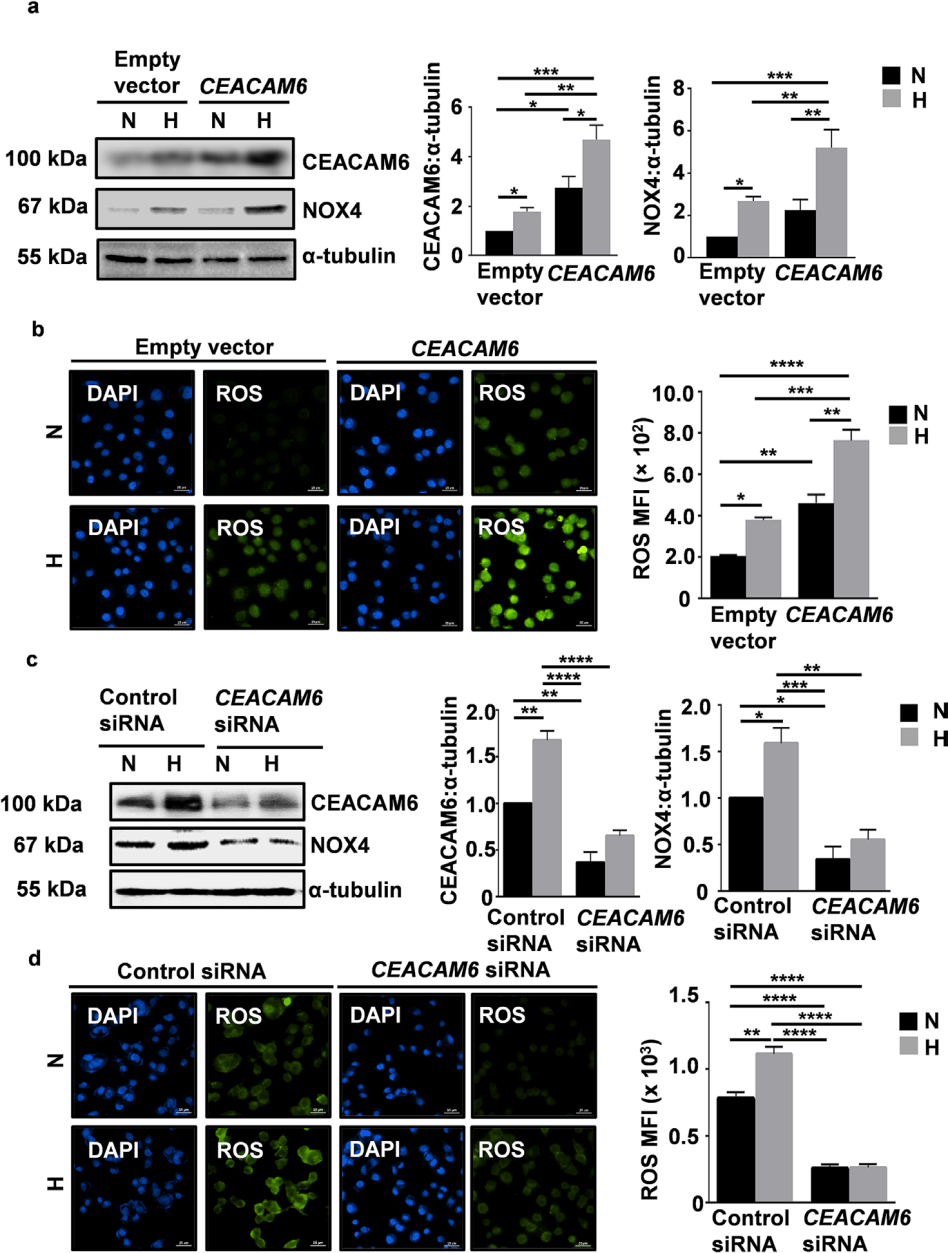
CEACAM6 upregulates NOX4 and increases ROS production in hypoxic AGS cells. (a) A representative western blot showing significant upregulation of NOX4 in *CEACAM6* stably expressing hypoxic AGS cells. (b) Immunofluorescence microscopy data showed elevated ROS production in *CEACAM6*‐stably expressing AGS cells in hypoxia. Bar graphs of MFI indicated a significant elevation of hypoxia‐mediated ROS generation in *CEACAM6*‐overexpressed stable AGS cells. (c) Hypoxia‐exposed, *CEACAM6* siRNA transiently transfected AGS cells analyzed by western blotting showed significant suppression of NOX4 protein levels. (d) Representative immunofluorescence microscopy performed on control and *CEACAM6* siRNA transiently transfected normoxic and hypoxic AGS cells and the MFI graph confirmed a significant reduction of hypoxia‐mediated ROS generation with the suppression of *CEACAM6*. In both b and d, nuclei were stained with DAPI. *n* = 3, Objective used 60×, scale bars represented 25 μm. All graphical data represented mean ± SEM. Two‐way ANOVA was performed and the results were corrected for multiple comparisons using Tukey's post hoc analysis. *n* = 3, *p < 0.05; **p < 0.01; ***p < 0.001; ****p < 0.0001.

To experimentally validate the findings from the bioinformatics studies and to study the importance of HIF1α regulation on CEACAM6 and NOX4, AGS cells with stable *HIF1α*‐suppression and the corresponding negative control cells [41] were exposed to normoxia or hypoxia. Whole‐cell lysates were western blotted. The result and its graphical representation showed a hypoxia‐mediated increase in CEACAM6 and NOX4, whereas those levels were significantly downregulated in *HIF1α*‐suppressed cells (Figure S4). To find the effect of *CEACAM6* overexpression on NOX4 regulation in hypoxia, the empty vector or *CEACAM6‐*overexpressing stable AGS cells were exposed to either hypoxia or normoxia. Western blot analysis and the accompanying graph revealed that NOX4 level increased significantly with *CEACAM6* overexpression (Figure 4a). To find out the effect of CEACAM6 on ROS regulation in hypoxia, *CEACAM6* or empty vector‐expressing stable AGS cells were exposed to hypoxia and normoxia followed by DCFDA treatment. A representative fluorescence microscopy result (*n* = 3) combined with the MFI graph showed significantly increased ROS generation in *CEACAM6*‐overexpressing cells which was more pronounced in cells exposed to hypoxia (Figure 4b). Western blot analysis further identified that siRNA‐mediated suppression of *CEACAM6* significantly downregulated NOX4 in AGS cells which again established the positive correlation between CEACAM6 and NOX4 (Figure 4c). Next, control siRNA and *CEACAM6* siRNA‐expressing cells were exposed to hypoxia or normoxia followed by detection of ROS using DCFDA. Representative fluorescence micrographs detected significant suppression of hypoxia‐induced ROS in CEACAM6‐suppressed cells compared with control siRNA‐expressing cells (Figure 4d).

### 3.5 NOX4 Mediates ROS Production in Hypoxic GECs

Next, to unravel the effect of *NOX4* overexpression on ROS generation in hypoxic and normoxic cells, empty vector and *NOX4*‐transfected (transient) AGS cells were exposed to hypoxia and evaluated for ROS signals using DCFDA. Fluorescence micrographs and the MFI graph confirmed *NOX4*‐mediated upregulation of ROS in hypoxia (Figure S5a). Accompanying western blot results showed the level of NOX4 in the cells used to generate the immunofluorescence micrograph. To further ascertain the role of NOX4 in ROS generation under hypoxia, control siRNA and *NOX4* siRNA transiently transfected AGS cells were exposed to hypoxia and normoxia followed by detection of ROS using DCFDA staining. siRNA‐mediated *NOX4* suppression significantly downregulated hypoxia‐induced ROS generation (Figure S5b). The paired western blot data showed the suppression of *NOX4* in the cells used in the immunofluorescence microscopy experiment.

### 3.6 Hypoxia Exposure and Hypoxia‐Reoxygenation of GECs Facilitate *H. pylori* Infection

**FIGURE 5:**
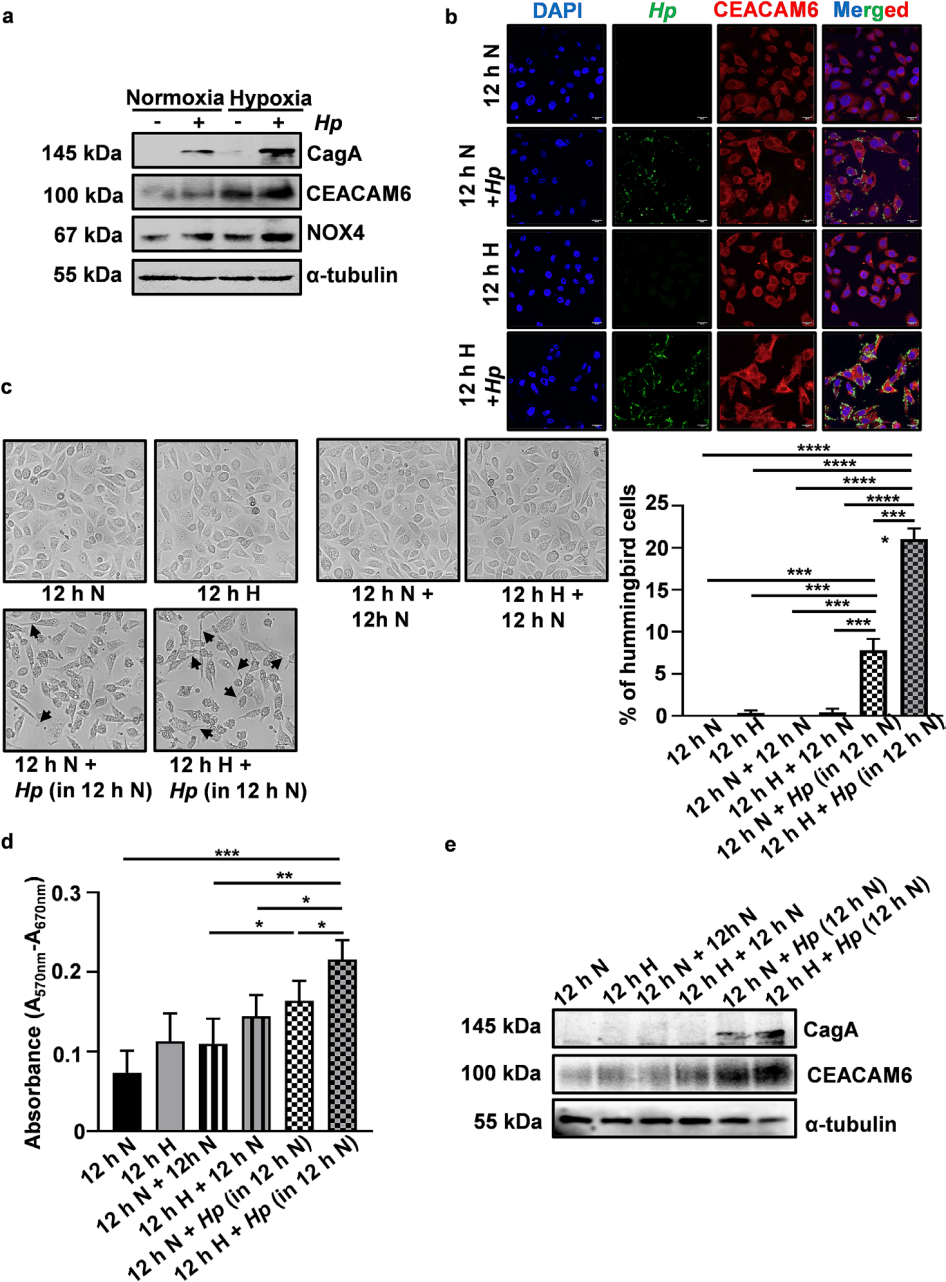
Hypoxia and hypoxia‐reoxygenation of GECs promote *H. pylori* infection. (a) CagA translocation, CEACAM6 and NOX4 protein levels in AGS cells increased in the presence of hypoxia and *H. pylori* (mentioned as *Hp* in the Figure) (graphical data can be found in Figure S6a–c). (b) Confocal imaging data revealed that *H. pylori* infection of AGS cells was increased in the presence of hypoxia. *H. pylori* = green, CEACAM6 = red. Nuclei were stained for DAPI. Objective used 63×, scale bar = 20 μm. (c) AGS cells were either kept in hypoxia or normoxia for 12 h followed by *H. pylori* (50 MOI) infection for 12 h or cells were left uninfected in normoxic condition. Bright field imaging (*n* = 3) revealed that hypoxia‐reoxygenated infected cells showed significantly increased hummingbird formation. Objective used 40×, scale bar = 25 μm. (d) Graphical representation of changes in proliferation of the same experimental sets of cells as mentioned in panel c and assessed by the MTT assay. The result showed significantly enhanced proliferation in hypoxia‐reoxygenated *H. pylori‐*infected cells as compared to the normoxia‐exposed infected cells. (e) A representative western blot (*n* = 3) of whole cell lysates from the same experimental sets showed a significant increase (graphical data shown in Figure S6d,e) in CagA translocation as well as CEACAM6 level in hypoxia‐reoxygenated infected cells as compared to the cells kept in normoxia before the infection. For all graphs = mean ± SEM. One‐way ANOVA was performed to assess the statistical significance between the different groups, *n* = 3, *p < 0.05; **p < 0.01; ***p < 0.001; ****p < 0.0001. In the Figure, *Hp =* *H. pylori* .

CEACAM6 promotes *H. pylori* adhesion to the GECs and *H. pylori* CagA cytotoxin upregulates CEACAM6 in early stages of GC [16, 54]. Considering this association of *H. pylori* pathogenesis with CEACAM6 expression, it was necessary to investigate whether hypoxia exposure and the resultant CEACAM6 upregulation play any role in CagA translocation in the GECs. For this, AGS cells were infected with *H. pylori* or left uninfected and incubated in normoxic or hypoxic conditions. Hypoxia‐exposed AGS cells showed a significant increase in CEACAM6 and NOX4 levels as well as CagA entry in cells (Figure 5a, Figure S6a–c). *H. pylori* adhesion to AGS cells was also noticeably higher when cells were exposed to hypoxia as compared to normoxia‐exposed cells (Figure 5b).

Fluctuating O_2_ conditions prevail in the gastrointestinal tract mucosa, including that of the stomach. Therefore, to assess the effect of hypoxia‐reoxygenation on *H. pylori* infection, AGS cells were exposed to either 12 h of hypoxia or normoxia followed by incubation at normoxia for 12 h in the presence or absence of *H. pylori* (MOI 50). It is worth mentioning here that GECs exposed to 12 h hypoxia followed by 12 h of *H. pylori* infection in hypoxia were not included in the study as GECs died under that condition. One of the prominent features of *H. pylori* infection and CagA translocation is the distinctive “hummingbird phenotype” [55]. To evaluate the hummingbird formation under hypoxia and hypoxia‐reoxygenation, cells were imaged by bright field microscopy for the identification of the “hummingbird phenotype.” The representative micrographs and graphical data (*n* = 3) showed that *H. pylori* ‐mediated “hummingbird” formation was significantly more in hypoxia‐reoxygenated cells than in the cells that were kept in normoxia (Figure 5c). The proliferation ability of AGS cells was analyzed using the MTT assay in the above‐mentioned experimental setup. Results demonstrated that hypoxia‐reoxygenated infected cells had significantly higher proliferation potential than normoxia‐exposed infected cells (Figure 5d). Interestingly, with a high CEACAM6 protein level in hypoxia‐reoxygenated AGS cells, the translocation of *H. pylori* CagA protein increased significantly when compared with normoxic cells (Figure 5e, Figure S6d,e).

### 3.7 *H. pylori* Infection is Promoted in CEACAM6‐Expressing Hypoxia‐Reoxygenated Cells

**FIGURE 6:**
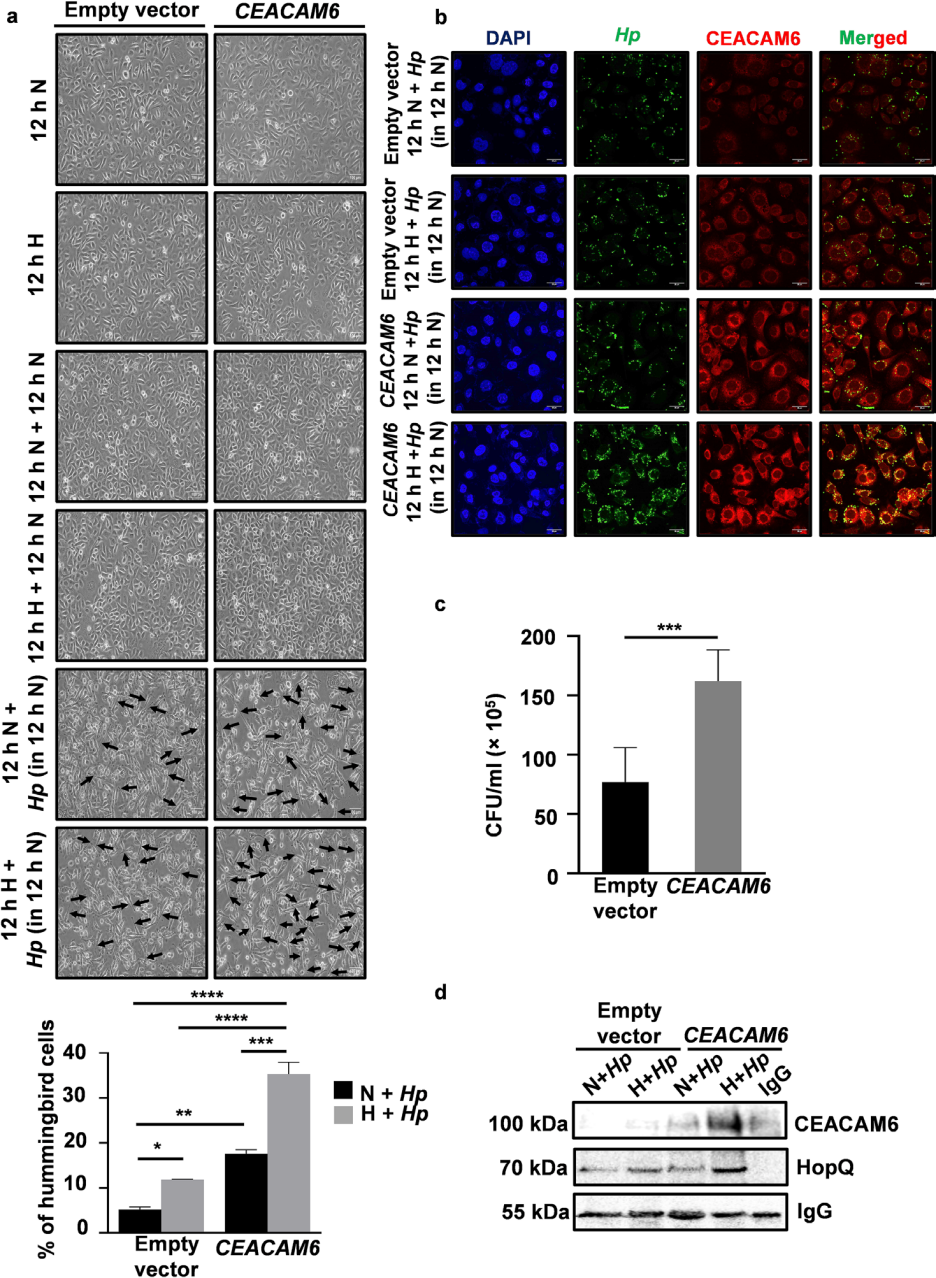
*Helicobacter pylori* infection is promoted in hypoxia‐reoxygenated *CEACAM6*‐expressing GECs. (a) Bright field micrographs and bar graph (*n* = 4) showing a significant increase in *H. pylori* ‐induced hummingbird formation in hypoxia‐reoxygenated and *CEACAM6* stably‐expressing AGS cells. Two‐way ANOVA was performed to assess the statistical significance between groups. Objective used 20X, scale bars representing 100 μm. (b) Confocal microscopy revealed that *H. pylori* adhesion to *CEACAM6* stably‐expressing hypoxia‐reoxygenated cells was noticeably more as compared to the empty vector‐expressing hypoxic cells. *H. pylori* = green, CEACAM6 = red. Nuclei were stained for DAPI. Objective used 63×, scale bars representing 20 μm. (c) Viable *H. pylori* count was significantly higher in *CEACAM6* overexpressed hypoxia and reoxygenation‐treated *H. pylori‐*infected cells compared to the empty vector‐expressing group. (d) Molecular complexes were prepared by coincubating bacterial outer membrane lysates and cell lysates from normoxic or hypoxic *CEACAM6* or empty vector‐expressing stable cells. These complexes were immunoprecipitated using CEACAM6 antibody and western blotted for HopQ and CEACAM6. IgG band ensured equal loading. For all graphs = mean ± SEM. Paired *t* test was performed to assess the statistical significance between the two groups, *n* = 3, *p < 0.05; **p < 0.01; ***p < 0.001; ****p < 0.0001. In the Figure, *Hp =* *H. pylori* .

**FIGURE 7:**
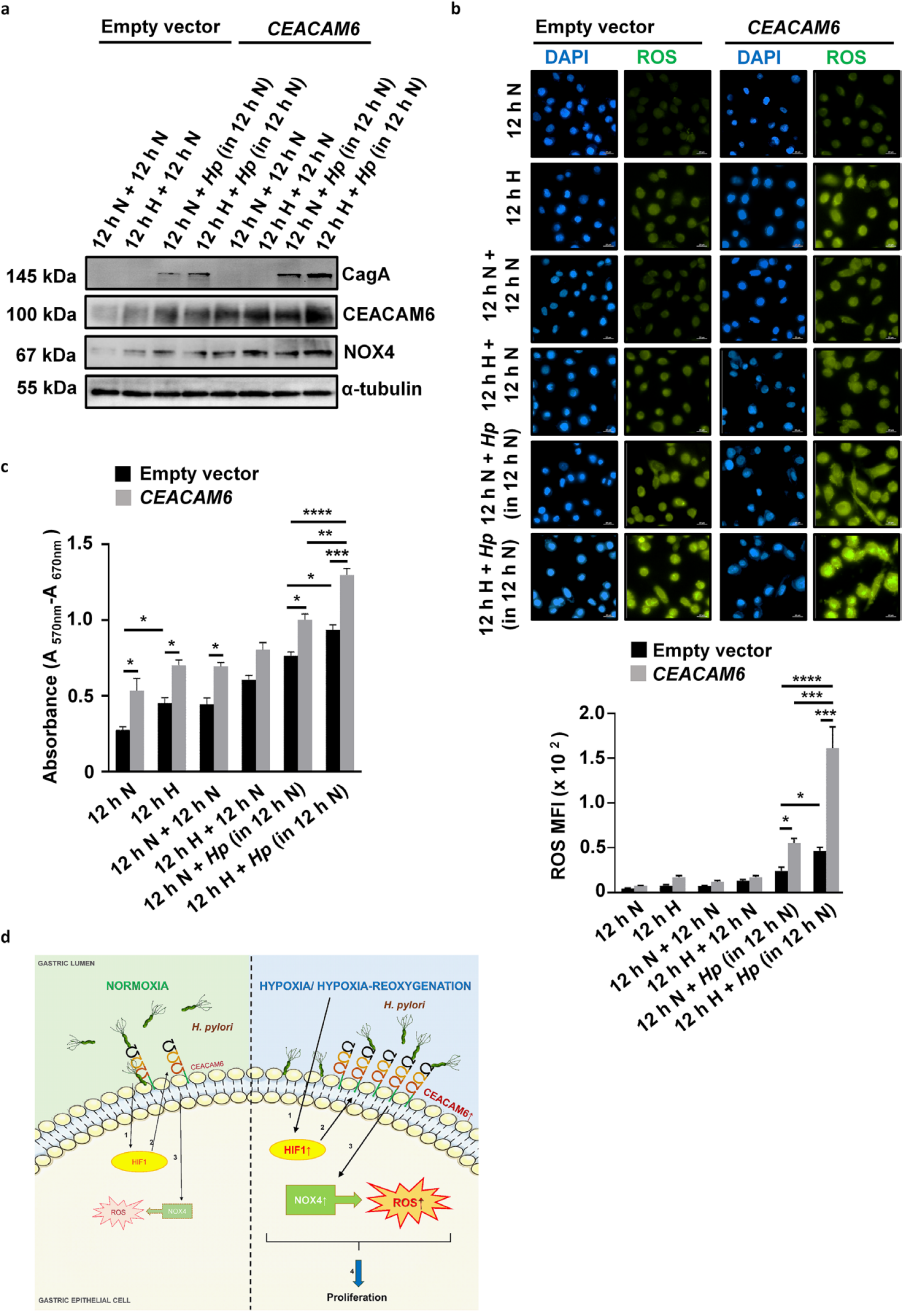
*CEACAM6*‐expressing GECs activate the CEACAM6‐NOX4 cascade, upregulate ROS generation, and gain significantly more proliferative ability after being exposed to hypoxia‐reoxygenation and *H. pylori* infection. (a) Western blot analysis showed that levels of CagA, CEACAM6, and NOX4 were significantly increased (graphical representations are shown in Figure S7a–c) in AGS cells upon *CEACAM6* stable overexpression and hypoxia‐reoxygenation before infection with *H. pylori* . (b) Fluorescence micrographs and graphical data (*n* = 3) demonstrated enhanced ROS production in hypoxia and reoxygenation‐exposed and *H. pylori* ‐infected *CEACAM6* overexpressing AGS cells when compared with the empty vector‐expressing stable cells with similar treatments. For ROS detection, cells were incubated with 1 μM DCFDA for 1 h after infection. Nuclei were stained with DAPI. (c) Graphical representation of cellular proliferation assessed by MTT assay (*n* = 3) showed significantly increased proliferation of *H. pylori* ‐infected cells with CEACAM6 overexpression as well as hypoxia‐reoxygenation when compared with the empty vector‐expressing as well as normoxia‐exposed cells. Objective used—60×, scale bars representing 25 μm. Graphs = mean ± SEM. Statistical significance was determined using two‐way ANOVA followed by Tukey's post hoc analysis (*n* = 3). *p < 0.05; **p < 0.01; ***p < 0.001; ****p < 0.0001. In the Figure, *Hp =* *H. pylori* . (d) The summary figure depicting the regulation of ROS signaling events in *H. pylori‐*infected normoxic and hypoxic GECs. *H. pylori* infection of GECs leads to the accumulation of HIF1α in normoxia, but the effect is far more enhanced in hypoxic cells due to the increased level of HIF1 and HIF1‐driven CEACAM6 upregulation. CEACAM6 facilitates *H. pylori* adhesion and upregulates ROS via enhanced NOX4 generation. Enhanced HIF1‐CEACAM6‐NOX4 signaling has a proliferative effect on GECs. Numbers indicate the sequence of events.

Once it is established that hypoxia‐reoxygenation promotes *H. pylori* infection in AGS cells, subsequent investigations examined whether CEACAM6 mediates the process. *CEACAM6*‐overexpressing or empty vector‐expressing stable cells were exposed to hypoxia‐reoxygenation followed by *H. pylori* infection. A representative bright field microscopy result (*n* = 3) and the summary graphical data showed that the percentage of hummingbird formation was significantly higher in the hypoxia‐reoxygenated *CEACAM6*‐expressing cells (Figure 6a). To find out whether CEACAM6 facilitates the binding of *H. pylori* with GECs, empty vector and *CEACAM6*‐expressing GECs were first exposed to hypoxia and then incubated with *H. pylori* in the normoxic condition. Confocal microscopy confirmed a noticeable increase in the binding of *H. pylori* with hypoxia‐ and reoxygenation‐exposed *CEACAM6*‐upregulated cells when compared with the empty vector‐expressing cells (Figure 6b). To further compare the *H. pylori‐*binding ability of *CEACAM6*‐expressing cells with empty vector‐expressing cells, they were exposed to hypoxia‐reoxygenation, infected with *H. pylori* , trypsinized, diluted, and streaked onto TSA plates for viable colony count. *H. pylori* count was significantly higher in hypoxia‐reoxygenated *CEACAM6*‐expressing cells as compared to their normoxic counterparts (Figure 6c). To correlate CEACAM6 status on GECs with *H. pylori* adhesion to cells, the outer membrane fraction of *H. pylori* was incubated with lysates of *CEACAM6*‐overexpressing stable cells experiencing normoxia/hypoxia or with respective controls, and the interacting protein complexes were pulled down using CEACAM6 antibody. Western blot analysis of the resulting immunocomplexes confirmed the interaction between CEACAM6 and HopQ, with the interaction getting stronger in hypoxic lysates with higher CEACAM6 content (Figure 6d). To find out whether CEACAM6‐mediated binding of *H. pylori* was accompanied by CagA translocation as well as NOX4 upregulation in hypoxia‐reoxygenated GECs or not, the empty vector‐expressing and *CEACAM6* stably‐expressing AGS cells were first incubated in hypoxia or normoxia followed by *H. pylori* infection in normoxia. A representative western blot (Figure 7a) and associated summary (*n* = 3) graphs showed a significant increase in CagA translocation and NOX4 level in the infected CEACAM6‐overexpressing hypoxia‐reoxygenated cells (Figure S7a–c). To find out whether hypoxia and *H. pylori* ‐mediated ROS generation was promoted by CEACAM6 or not, *CEACAM6*‐overexpressing or empty vector‐expressing AGS cells were exposed to hypoxia‐reoxygenation or normoxia followed by *H. pylori* infection and DCFDA treatment. Immunofluorescence microscopy was performed and the fluorescence intensity was measured. The representative results and the summary plot (*n* = 3) confirmed that hypoxia‐reoxygenation significantly induced ROS generation in *H. pylori* ‐infected *CEACAM6*‐expressing AGS cells when compared with normoxic empty vector‐expressing *H. pylori* ‐infected AGS cells (Figure 7b). To find out the proliferation ability of hypoxia‐reoxygenated empty vector‐expressing and *CEACAM6*‐expressing stable cells after *H. pylori* infection, MTT assay was performed. The results corroborated that *CEACAM6* expression added more proliferative potential to the hypoxia‐reoxygenated cells that were later infected with *H. pylori* as compared to the cells of the empty vector‐expressing group (Figure 7c). Figure 7d summarily describes the findings of this study.

## 4 Discussion

*Helicobacter pylori* infection initiates neoplastic alterations in GECs. The colonization of the pathogen is influenced by CEACAM proteins. This study shows for the first time that hypoxia‐mediated CEACAM6 increase in GECs is retained upon reoxygenation and it augments ROS generation by upregulating NOX4. Interestingly, hypoxia‐exposed GECs, due to their elevated CEACAM6 level, ensure higher *H. pylori* adhesion, CagA translocation, and NOX4 upregulation. Successive hypoxia and reoxygenation‐exposed CEACAM6‐expressing GECs show significantly increased CagA translocation, hummingbird formation, ROS generation, and proliferation ability driven by *H. pylori* when compared with GECs that are not exposed to hypoxia before the infection. These results collectively confirm that hypoxia and reoxygenation make gastric epithelium more suitable for *H. pylori* colonization and infection, thus increasing the risk of GC.

Physiological hypoxia is common in the human gastrointestinal tract due to perfusion fluctuations, and the human stomach too is not an exception [28]. Pathological hypoxia ensues in respiratory or circulatory illnesses, infections, inflammatory events, and inside solid tumors; and like physiological hypoxia, pathological hypoxia also stabilizes HIF1α [11]. This work identifies that HIF1α‐mediated CEACAM6 upregulation in hypoxic GECs enhances cellular ROS load by involving NOX4. This mechanism of ROS generation is retained in the reoxygenated gastric epithelium, and eventual *H. pylori* infection further potentiates ROS accumulation. This finding is very crucial to understanding various physiological and pathological conditions that involve intermittent hypoxia in the gastric epithelium. Literature shows that both hypoxia‐ and hypoxia‐reoxygenation events are largely driven by HIF1 [56]. Interestingly, HIF1 induces several bacterial infections in the gastrointestinal and respiratory tract [57]. Increased bacterial adhesion to the airway epithelium is associated with hypoxia‐mediated HIF1 activation [58]. There is evidence of hypoxia and HIF‐mediated host responses that aid in pathogen binding in human airway epithelial cells [58, 59]. HIF1 regulates *E. coli* infection in Crohn's disease through CEACAM6 [60]. Since hypoxia and reoxygenation of GECs significantly promote *H. pylori* binding, CagA translocation, and ROS generation via CEACAM6 upregulation, it can be speculated that HIF1‐mediated CEACAM6 upregulation might be a critical determinant of successful bacterial infection.

*Helicobacter pylori* CagA protein can upregulate CEACAM6 [16]. CEACAM6 facilitates CagA delivery to the GECs along with other CEACAM proteins [61]. Our observation of CEACAM6‐mediated augmented binding of *H. pylori* to hypoxic GECs provides crucial information regarding the adhesion of not only *H. pylori* but also many CEACAM‐interacting pathogenic bacteria. It is also important to note that *H. pylori* ‐directed cell proliferation is enhanced in hypoxic and hypoxia‐reoxygenated GECs, thus reiterating the importance of hypoxia in gastric carcinogenic events. Here, it is important to remember that CEACAM6 is a raft‐associated glycophosphatidylinositol (GPI)‐linked membrane protein [62, 63]. Interestingly, translocation to lipid rafts is suggested to be required for NOX4 activation in adipocytes experiencing high glucose and palmitate [64]. Although the existing literature does not describe the impact of hypoxia in regulating the localization of NOX4 in lipid rafts, the transmembrane topology of NOX4 indicates that both the N‐ and C‐termini of the protein face the cytosol [65]. This revelation projects NOX4 as a good signaling partner. The strength of this study lies in the fact that it unravels a hitherto unknown phenomenon that prior hypoxia exposure or hypoxia‐reoxygenation facilitates *H. pylori* infection. Moreover, it also highlights the importance of the HIF1‐CEACAM6‐NOX4 axis in the regulation of ROS in the gastric epithelium. However, further investigation is needed to gain in‐depth knowledge about the membrane‐proximal signaling partners of CEACAM6 involved in activating NOX4 in hypoxia, hypoxia‐reoxygenation, and *H. pylori* ‐driven GC.

In conclusion, these results highlight the significance of CEACAM6 upregulation in *H. pylori* infection, successful colonization in the hypoxia‐exposed and hypoxia‐reoxygenated gastric epithelial tissue, and establishment of GC by inducing cell proliferation. As ROS upregulation by NOX4 is critically regulated by CEACAM6, we find that CEACAM6 and NOX4 have great potential as diagnostic and prognostic biomarkers of GC involving *H. pylori* infection and hypoxia.

## Author Contributions

**Indrajit Poirah:** data curation (lead), formal analysis (lead), methodology (lead), validation (lead), visualization (lead), writing – original draft (lead), writing – review and editing (lead). **Soumyadeep Chakraborty:** formal analysis (supporting). **Pratyush Kumar Padhan:** formal analysis (supporting). **Ashish Kumar Mishra:** formal analysis (supporting). **Debashish Chakraborty:** writing – review and editing (supporting). **Pragyesh Dixit:** writing – review and editing (supporting). **Supriya Samal:** writing – review and editing (supporting). **Niranjan Rout:** resources (supporting). **Shivaram Prasad Singh:** resources (supporting). **Gautam Nath:** resources (supporting). **Duane T. Smoot:** resources (supporting). **Hassan Ashktorab:** resources (supporting). **Asima Bhattacharyya:** conceptualization (lead), formal analysis (lead), investigation (lead), project administration (lead), supervision (lead), writing – original draft (supporting), writing – review and editing (lead).

## Ethics Statement

Human tissue collection was approved by the Institutional Ethics Committee for Human Research, National Institute of Science Education and Research (NISER) (protocol No. NISER/IEC/2018‐01).

## Consent

Prior written consent from the patients was obtained.

## Conflicts of Interest

The authors declare no conflicts of interest.

## Supporting information


Data S1

## Data Availability

The data that support the findings of this study are available from the corresponding author upon reasonable request.
