# Supplementary material for: Hypoxia and Hypoxia‐Reoxygenation Potentiate Helicobacter pylori Infection and Gastric Epithelial Cell Proliferation
Source: Cancer Med. 2025 May 5;14(9):e70860. doi: 10.1002/cam4.70860 (PMC12053057; doi:10.1002/cam4.70860)
Supplement: Supplementary file 1 — Data S1 [file CAM4-14-e70860-s001.pdf]

Online Resource 1: Supplementary Figures

Hypoxia and hypoxia-reoxygenation potentiate *Helicobacter pylori* infection and gastric epithelial cell proliferation

Indrajit Poirah[1], Soumyadeep Chakraborty[1], Pratyush Kumar Padhan[1], Ashish Kumar Mishra[1], Debashish Chakraborty[1], Pragyesh Dixit[1], Supriya Samal[1], Niranjan Rout[2], Shivaram Prasad Singh[2], Gautam Nath[3], Duane T. Smoot[4], Hassan Ashktorab[5], Asima Bhattacharyya[1,6]

Affiliations: [1]School of Biological Sciences, National Institute of Science Education and Research (NISER) Bhubaneswar, An OCC of Homi Bhabha National Institute, P.O. Bhimpur-Padanpur, Via Jatni, Khurda 752050, Odisha, India; [2]Digestive Diseases Centre, Beam Diagnostics Building, Bajrakabati Road [Shanti Nagar], Cuttack 753001, Odisha, India; [3]Department of Gastroenterology, Acharya Harihar Post Graduate Institute of Cancer, Cuttack 753007; [4]Department of Medicine, Meharry Medical Center, Nashville, TN, 37208, USA; [5]Department of Medicine, Howard University, Washington DC, 20060, USA; [6]Centre for Interdisciplinary Sciences (CIS), NISER, An OCC of Homi Bhabha National Institute, P.O. Bhimpur-Padanpur, Via Jatni, Khurda 752050, Odisha, India.

Correspondence: School of Biological Sciences, National Institute of Science Education and Research (NISER) Bhubaneswar, An OCC of Homi Bhabha National Institute, P.O. Bhimpur-Padanpur, Via Jatni, Khurda, 752050, Odisha, India. Tel: +91-674-2494210, Email: asima@niser.ac.in

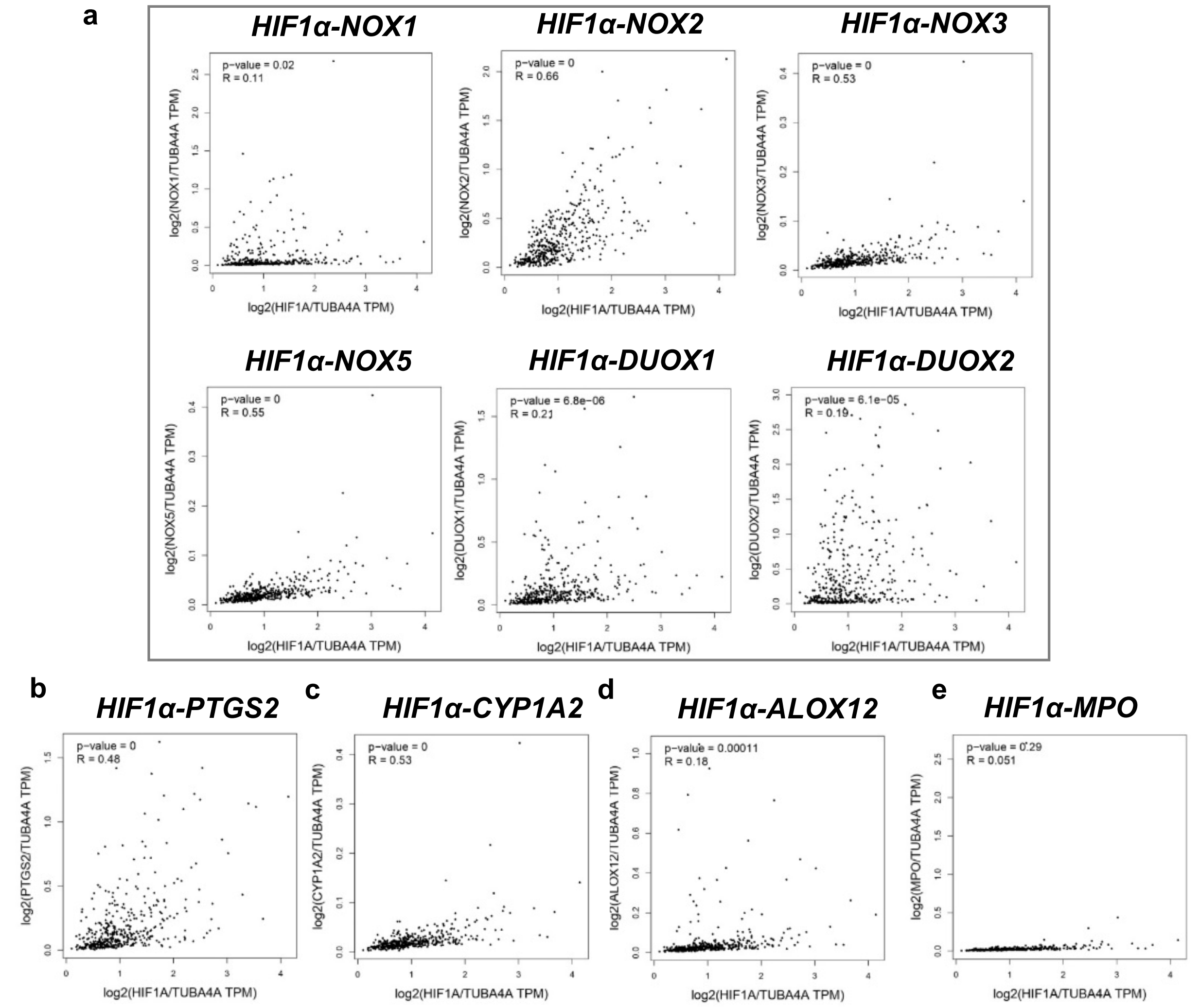

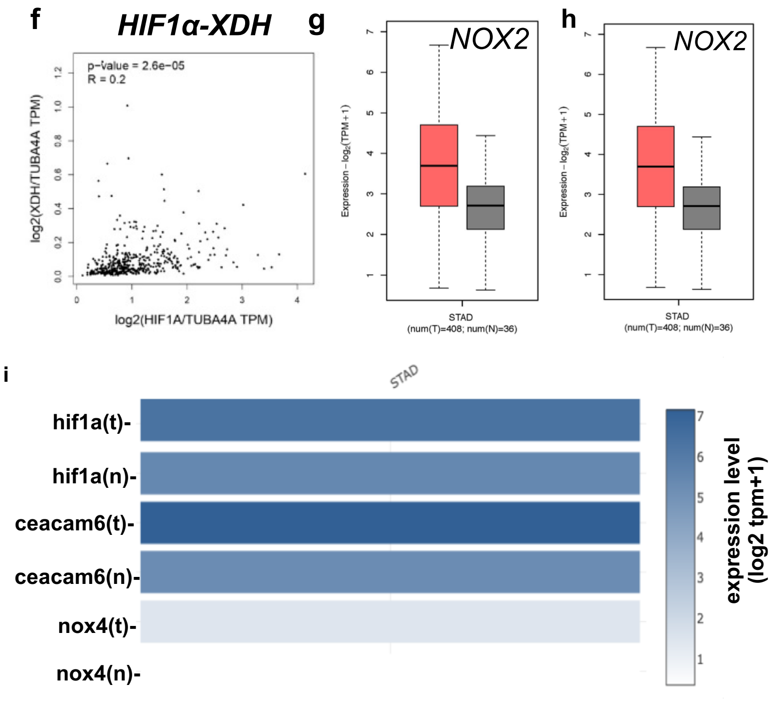

Figure S1 (a) Pearson's correlation coefficient between normalized *HIF1α* mRNA expression level and that of *NOX* family members in STAD tissue performed using GEPIA2 with data curated from the TCGA dataset. (b-f) Pearson's correlation coefficient between *HIF1α* and other ROS generating enzymes. (g) Box plot representation of *NOX2* expression in STAD tumor (red) and normal cells (grey) {num(T)=408, num(N)=36, *p* value cut off set at 0.01} and (h) at *p* value cut off set at 0.05 does not show any significant difference. (i) Multiple gene comparison of *HIF1α*, *CEACAM6* and *NOX4* show their higher expression in STAD tumor as compared to STAD normal tissue.

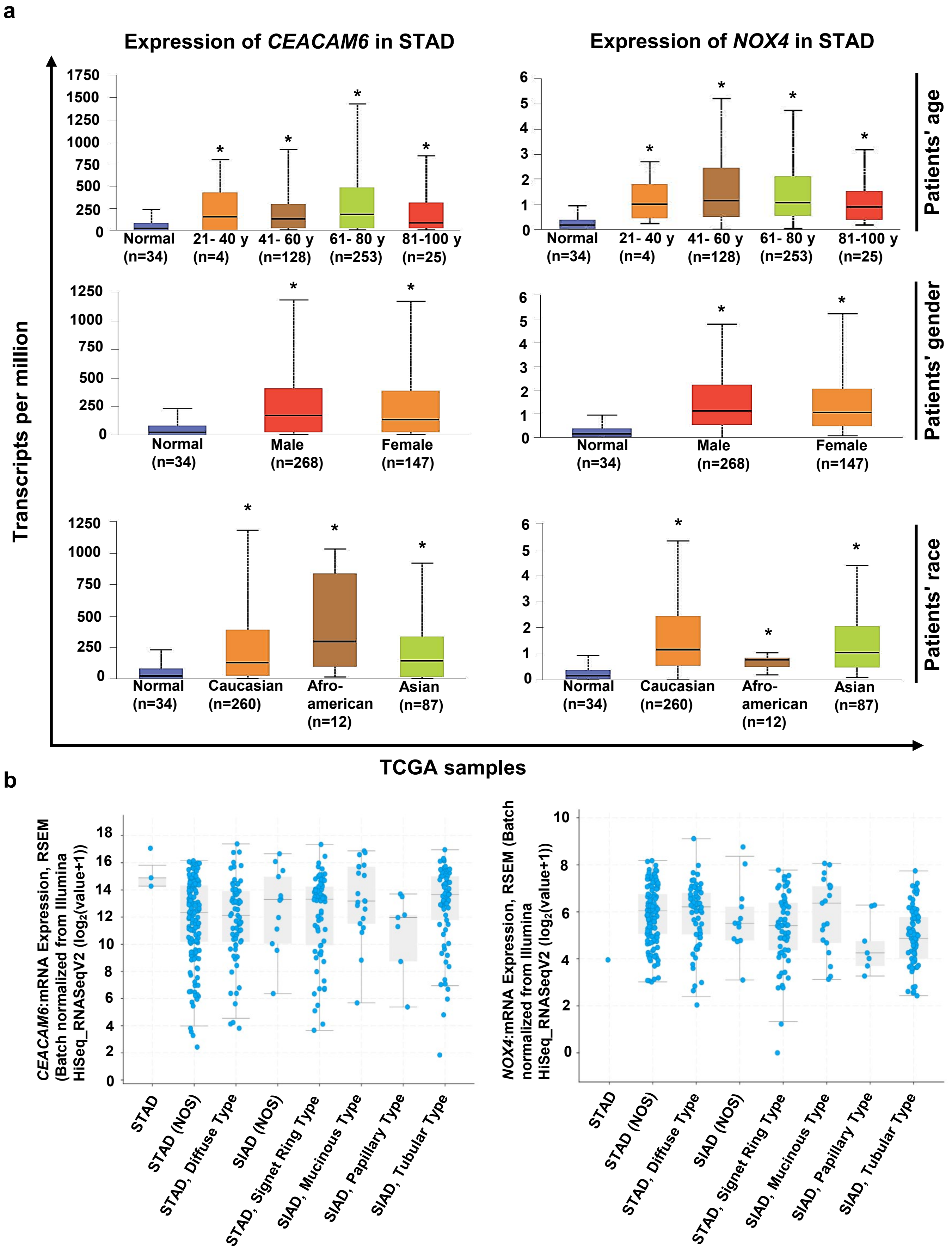

Figure S2 (a) Expression profiles of *CEACAM6* and *NOX4* genes in TCGA STAD based on the patient's age, gender and race curated from the UALCAN database. In all cases, various STAD patients have significantly higher expression of *CEACAM6* and *NOX4* than normal. (b) *CEACAM6* and *NOX4* mRNA expression across various STAD types as analysed by cBioPortal. STAD = Stomach Adenocarcinoma, SIAD = Stomach Intestinal Adenocarcinoma. NOS = not otherwise specified. In panel a, * indicates significance.

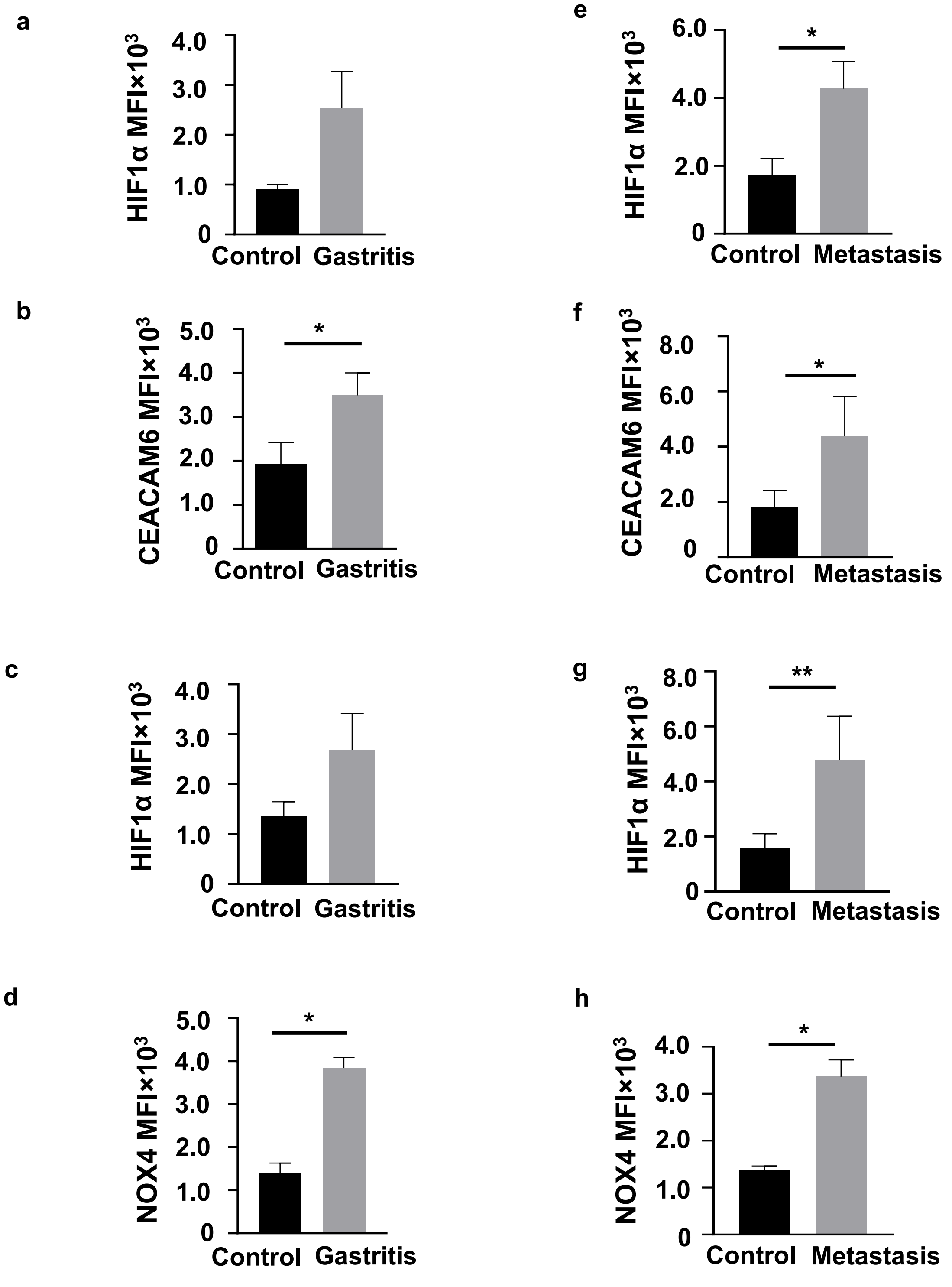

Figure S3 Bar graphs accompanying **Figure 3f** showing significantly enhanced CEACAM6 (b), NOX4 (d) and increased HIF1α (a,c) levels in paired control and gastritis patient samples. Graphs accompanying **Figure 3g** show a significant increase in HIF1α (e, g), CEACAM6 (f) and NOX4 (h) levels in paired control and metastatic tissues. Data represent mean ± SEM. Student's t-test was performed for statistical measurements followed by Tukey's post hoc analysis, n=3, * $p < 0.05$, ** $p < 0.005$.

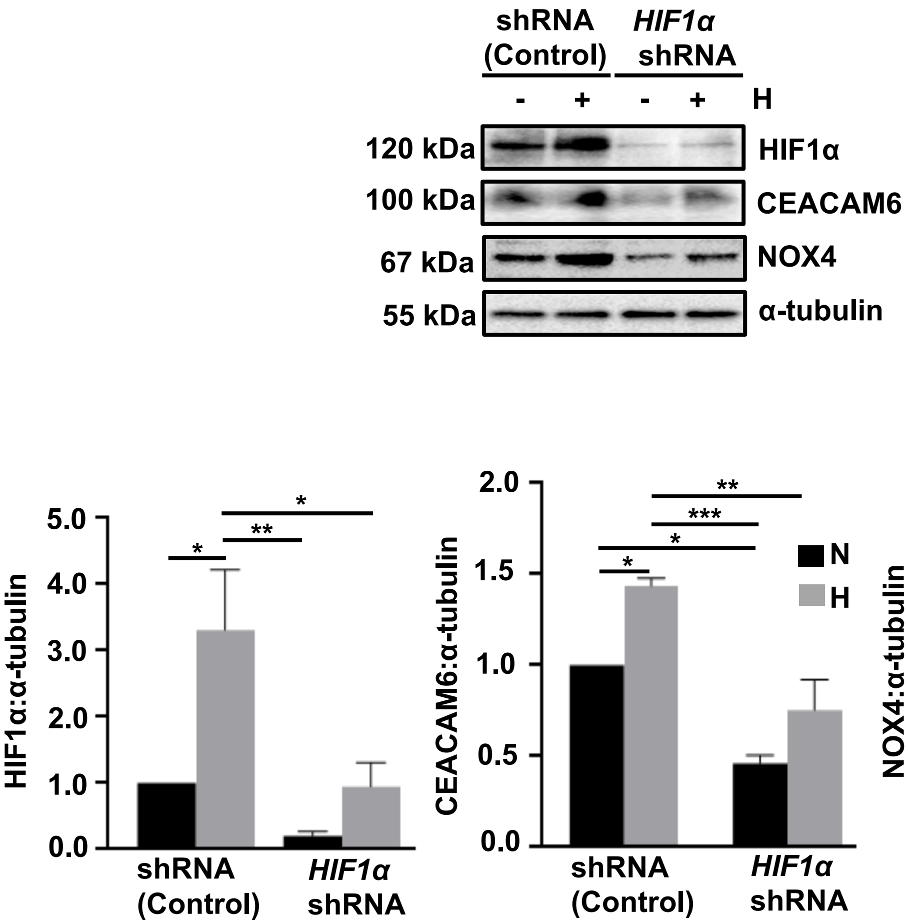

Figure S4 HIF1α regulates NOX4 and CEACAM6 in AGS cells. Western blot analyses were performed on whole cell extracts of hypoxia or normoxia-exposed *HIF1α* shRNA-transfected as well as control shRNA-transfected AGS cells. HIF1α, NOX4 and CEACAM6 protein levels were determined. Bar graphs show a significant increase in HIF1α, NOX4 and CEACAM6 levels with hypoxia, whereas a significant reduction is seen in the absence of HIF1α. N= normoxia, H= hypoxia. Graphical data indicates mean ± SEM. Two-way ANOVA was performed to determine the statistical significance and the results were corrected for multiple comparisons using Tukey's post-hoc analysis. n=3, * p < 0.05; **p < 0.01; ***p < 0.001.

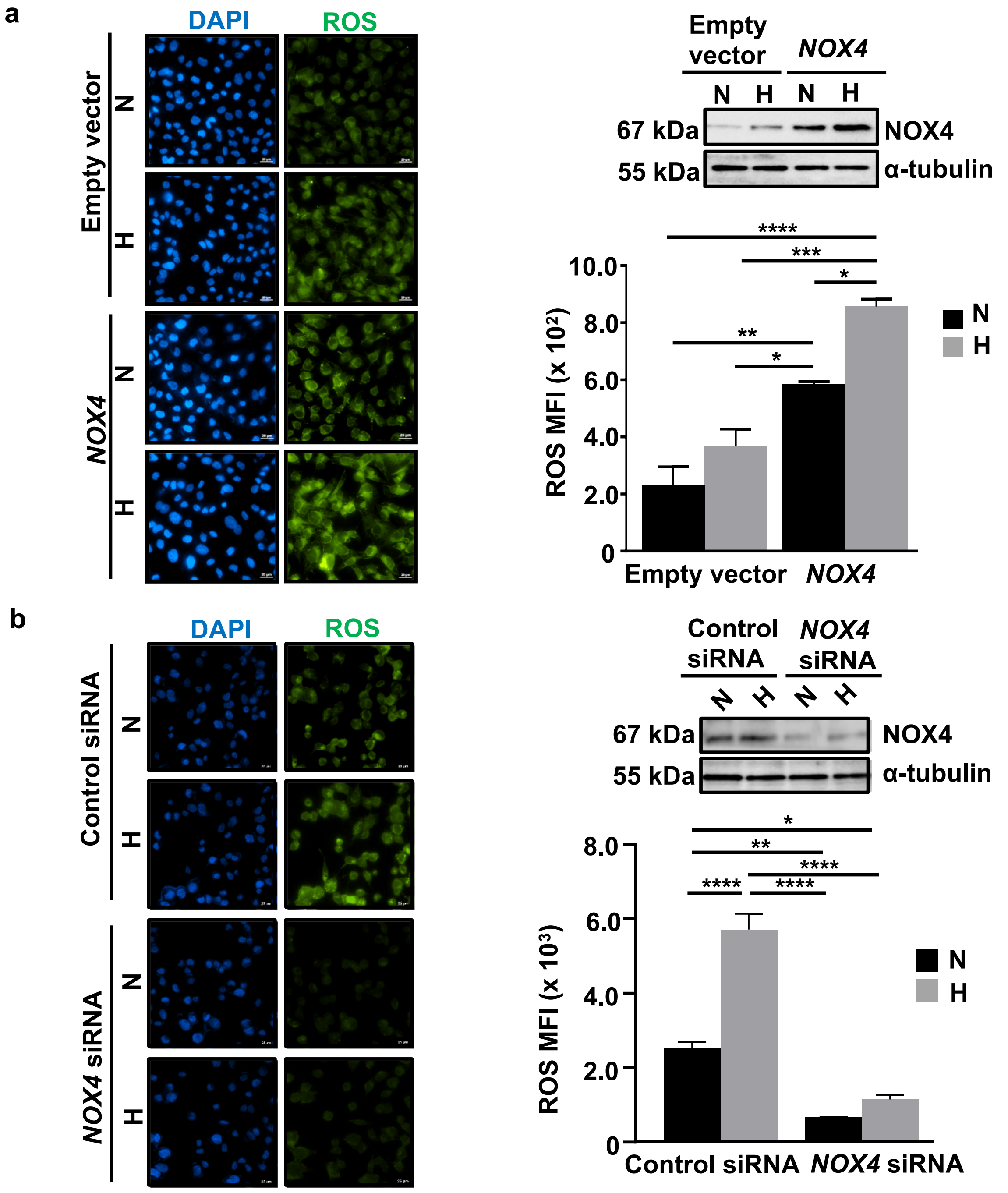

Figure S5 Cellular ROS production in hypoxia is dependent on NOX4. (a) Immunofluorescence microscopy data accompanied by MFI graphs show that hypoxia-mediated ROS generation is significantly enhanced in NOX4 transiently-transfected AGS cells. The accompanying western blot shows the NOX4 overexpression status. Objective used 60X, scale bars = 20 μm. (b) A representative fluorescence micrograph shows significantly reduced hypoxia-mediated ROS generation in *NOX4*-suppressed AGS cells. Objective used 60X, scale bars = 25 μm. Accompanying western blot of whole cell extracts of *NOX4* siRNA transiently-transfected cells show NOX4 suppression at the protein level. All graphical data indicate mean ± SEM. Two-way ANOVA was used to measure the statistical significance of the data followed by Tukey's post hoc analysis, n=3, *$p < 0.05$; **$p < 0.01$; ***$p < 0.001$; ****$p < 0.0001$.

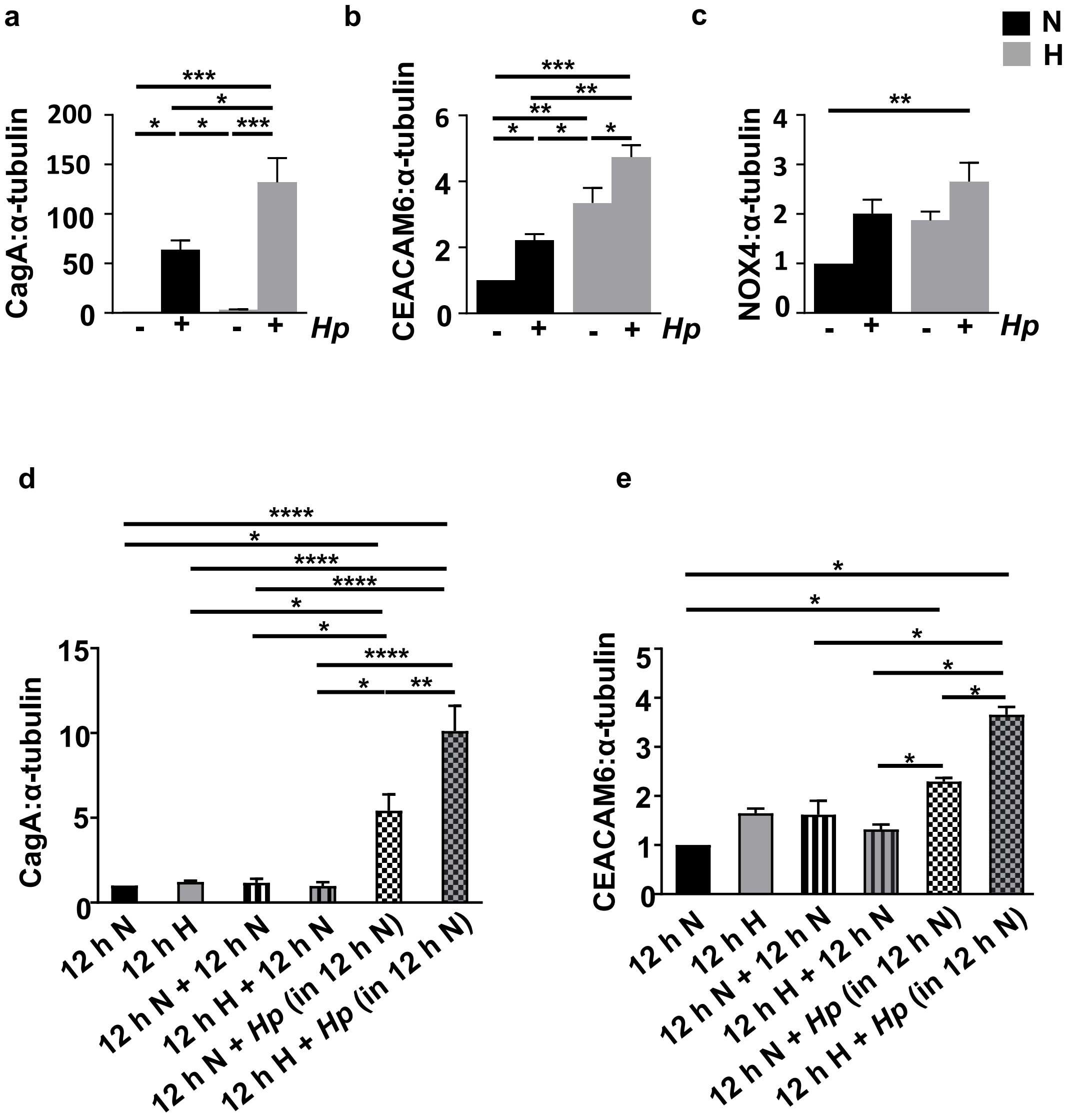

Figure S6 Bar graphs accompanying **Figure 5a** indicating the increase in CagA transfer and increase in CEACAM6 levels in the presence of hypoxia as well as *H. pylori* and changes in NOX4 levels when both hypoxia and *H. pylori* were present together. Graphs accompanying **Figure 5e** indicating the increase in CagA translocation and CEACAM6 levels in AGS cells exposed to hypoxia followed by *H. pylori* infection. Graphs show a significant increase in **d** CagA:α-tubulin and **e** CEACAM6:α-tubulin levels in *H. pylori*-infected AGS cells previously exposed to hypoxia compared to those kept in normoxic conditions. Graphical data indicate mean ± SEM. One-way ANOVA followed by Tukey's post hoc analysis was performed for statistical evaluation, n=3, *$p < 0.05$; **$p < 0.01$; ***$p < 0.001$ ****$p < 0.0001$.

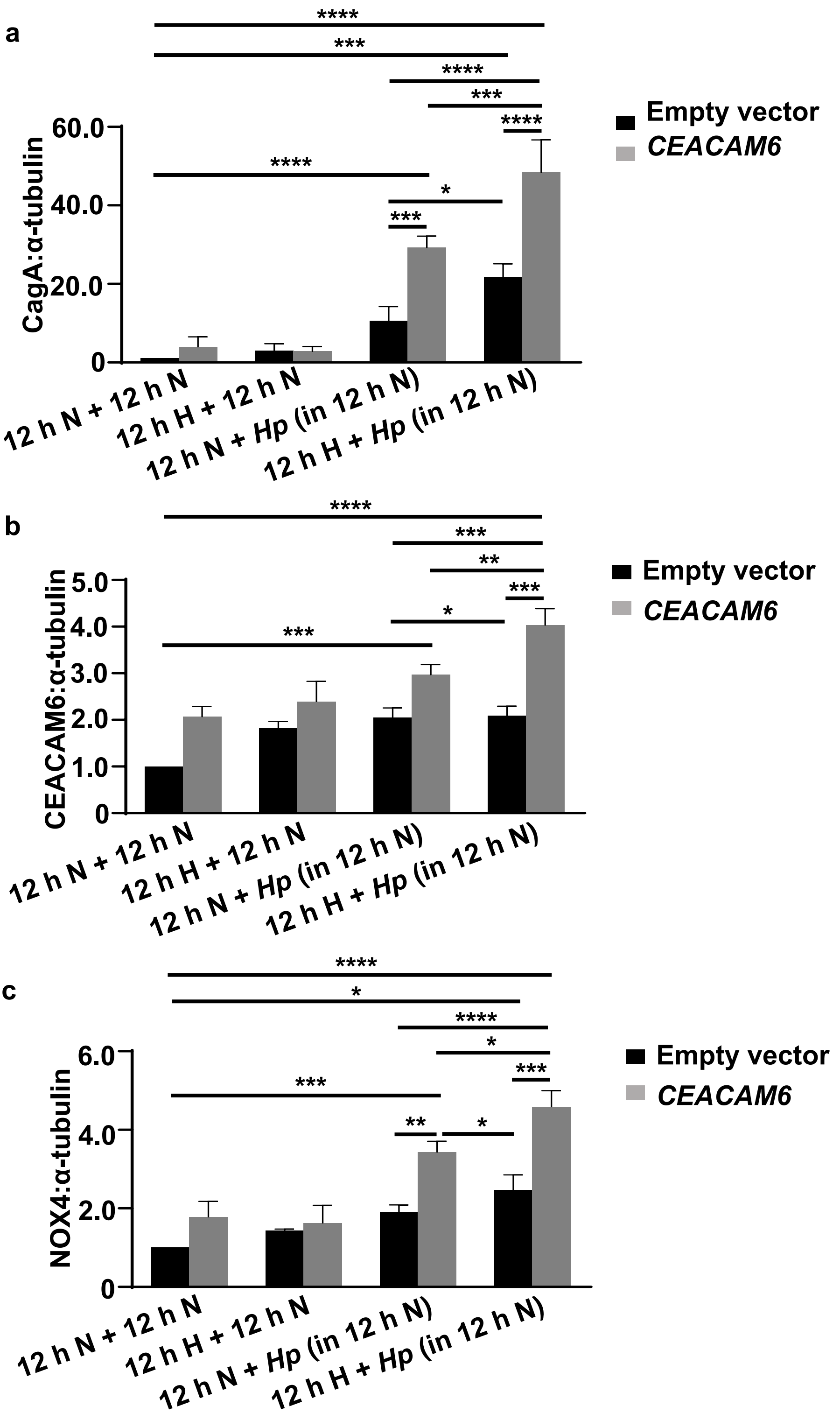

Figure S7 Bar graphs accompanying **Figure 7a** indicate significantly enhanced CagA translocation and cellular NOX4 levels in *CEACAM6*-expressing, hypoxia pre-exposed AGS cells following *H. pylori* infection. Graphs show significant increase in (a) CagA:α-tubulin, (b) CEACAM6:α-tubulin and (c) NOX4:α-tubulin levels in *H. pylori*-infected AGS cells pre-exposed to hypoxia compared to those kept in normoxic condition. Graphical data indicate mean ± SEM. Two-way ANOVA was performed followed by Tukey's post hoc analysis, n=3, * $p < 0.05$; ** $p < 0.01$; *** $p < 0.001$; **** $p < 0.0001$.
